# Modular robotic platform for precision neurosurgery with a bio-inspired needle: System overview and first *in-vivo* deployment

**DOI:** 10.1371/journal.pone.0275686

**Published:** 2022-10-19

**Authors:** Riccardo Secoli, Eloise Matheson, Marlene Pinzi, Stefano Galvan, Abdulhamit Donder, Thomas Watts, Marco Riva, Davide Danilo Zani, Lorenzo Bello, Ferdinando Rodriguez y Baena

**Affiliations:** 1 The Mechatronics in Medicine Lab, Department of Mechanical Engineering, Imperial College London, London, United Kingdom; 2 Department of Biomedical Sciences, Humanitas University, Milan, Italy; 3 Istituto di Ricovero e Cura a Carattere Scientifico Humanitas Research Hospital Rozzano, Rozzano, Italy; 4 Department of Veterinary Medicine, Universitá degli Studi di Milano, Lodi, Italy; 5 Department of Oncology and Hematology-Oncology, Universitá degli Studi di Milano, Milan, Italy; Istituto Italiano di Tecnologia, ITALY

## Abstract

Over the past 10 years, minimally invasive surgery (MIS) has shown significant benefits compared to conventional surgical techniques, with reduced trauma, shorter hospital stays, and shorter patient recovery times. In neurosurgical MIS procedures, inserting a straight tool (e.g. catheter) is common practice in applications ranging from biopsy and laser ablation, to drug delivery and fluid evacuation. How to handle tissue deformation, target migration and access to deep-seated anatomical structures remain an open challenge, affecting both the preoperative planning phase and eventual surgical intervention. Here, we present the first neurosurgical platform in the literature, able to deliver an implantable steerable needle for a range of diagnostic and therapeutic applications, with a short-term focus on localised drug delivery. This work presents the system’s architecture and first *in vivo* deployment with an optimised surgical workflow designed for pre-clinical trials with the ovine model, which demonstrate appropriate function and safe implantation.

## Introduction

Minimally invasive surgery (MIS) has seen significant growth over the last 10 years [1]. Rapid developments have taken place due to the significant benefits MIS presents for the patient, including less trauma, shorter hospital stays and reduced recovery times, as reported by [2]. Catheter insertions are highly prevalent in both diagnostic and interventional MIS neurosurgical procedures, including biopsy, ablation, brachytherapy and fluid delivery and extraction. The success of these procedures is dependent on the precision and accuracy of the tip placement at the target position and orientation (i.e. the pose). These procedures can be aided by robotic steerable, flexible catheter systems [3], as such devices can reach a given target precisely, increase the reachable workspace of the catheter tip and avoid critical areas or obstacles, such as nerves, vessels and bones [4, 5].

In soft tissue, flexible catheters follow paths which are affected by deformation/distortion resulting from inhomogeneity and anisotropy of the tissue, organ deformation and physiological processes including but not limited to respiration, brain shift and swelling [6, 7]. Active steering can mitigate misalignment that may be the result of operator error, catheter deflections and dynamic soft tissue interactions, as reviewed comprehensively in [8]. The development of steerable needles will also increase the number of procedures that can be performed via MIS, as these catheters will be able to reach targets that were previously occluded by complicated geometry [9, 10].

Several needle steering technologies have been developed to provide curvilinear paths within tissue. These can be broadly classified into seven main categories: needle steering controlled using concentric tubes, also known as active cannulas [11–13], needle steering controlled using the lateral motion of the needle base [14, 15], flexible needle steering controlled using a fixed shaped bevel tip (with and without pre-curve) [7, 16–21], pre-curved stylets [22], tendon actuated tips [23], optically controlled needles [24] and flexible needle steering controlled using a bio-inspired multi-segment design or programmable bevel-tip (PBN) [25, 26]. Since then, shape memory alloy (SMA) actuated flexible needles [27, 28] and magnetic driven needles [29] have also been presented. Neurosurgery is a field that can greatly benefit from robotic solutions [30–32], not least because of the rich history of neurosurgical innovation in stereotaxy, a constrained anatomical environment, the microsurgical nature of procedures, a highly technical nature of the field, a need for growth in MIS and a culture that adopts and embraces new technology [33]. However, general system solutions are rare, likely due to the inherently complex nature of procedures. Early robotic neurosurgical platforms served as computer-assisted stereotactic guidance systems. Indeed, the first medical robotic demonstration in 1985 used a PUMA 560 Industrial robot to guide a brain biopsy needle to a target along a straight trajectory [34]. In 1991, a later version of the system allowed the successful resection of deep benign astrocytomas in 6 children without morbidity or mortality [35].

Currently, the Renishaw neuromate^®^ stereotactic robot is a commercially available 5 Degrees of Freedom (DoF) serial manipulator system [36] suitable for a broad range of procedures such as deployment of electrodes for Deep Brain Stimulation (DBS) [37], stereo-electroencephalography [38] and other stereotactic applications. Other robotic, frameless stereotactic solutions include the Zimmer Biomet (originally MedTech) Rosa brain, a 6 DoF serial robotic manipulator designed for the accurate placement and insertion of neurosurgical tools [39], the Medtronic (originally Mazor Robotics) Renaissance system, a hexapod parallel robotic manipulator with 6 DoF that is directly mounted to the skull of the patient and used for DBS and biopsies, and CyberKnife [40]. CyberKnife is a frameless platform for stereotactic radiosurgery—a non-invasive procedure that uses precisely targeted radiation as an ablative surgical tool. It consists of a 6DoF arm that points the medical linear accelerator (LINAC) using real-time image guidance. Software interfaces for catheter insertion tasks combine pre-operative medical imaging and intraoperative sensing coupled with optional imaging (such as ultrasound, US). Using processed Magnetic Resonance Imaging (MRI) and Computerised Tomography (CT) scans, neurosurgeons can see depictions of the 3D brain volume, and navigate through the anatomy via 2D views in the Axial, Coronal and Sagittal planes [41]. However, it can be challenging to correlate 2D slices to a 3D track [42]; hence interfaces for steerable needles need to be redesigned for intuitive control. One method is to augment the view with overlays [43, 44].

Different visual interfaces have been presented for various applications. A visual interface for steering of magnetic micro-agents using US and other slow 2D imaging modalities was presented in [45]. Overlays tracking a target in 3D using US imaging were presented in [46], and a a visual interface for catheter insertions in lung and liver rendered as a 3D interface by using CT imaging was presented in [47, 48]. Existing robotic neurosurgical platforms can undertake various procedures, including instrument delivery, resection, and electrode implantation. However, to the best knowledge of the authors, there is currently a limited number of existing pre-commercial solutions for the delivery and control of steerable needles [48, 49], in particular programmable bevel-tip needles, none for neurosurgical procedures.

**Fig 1:**
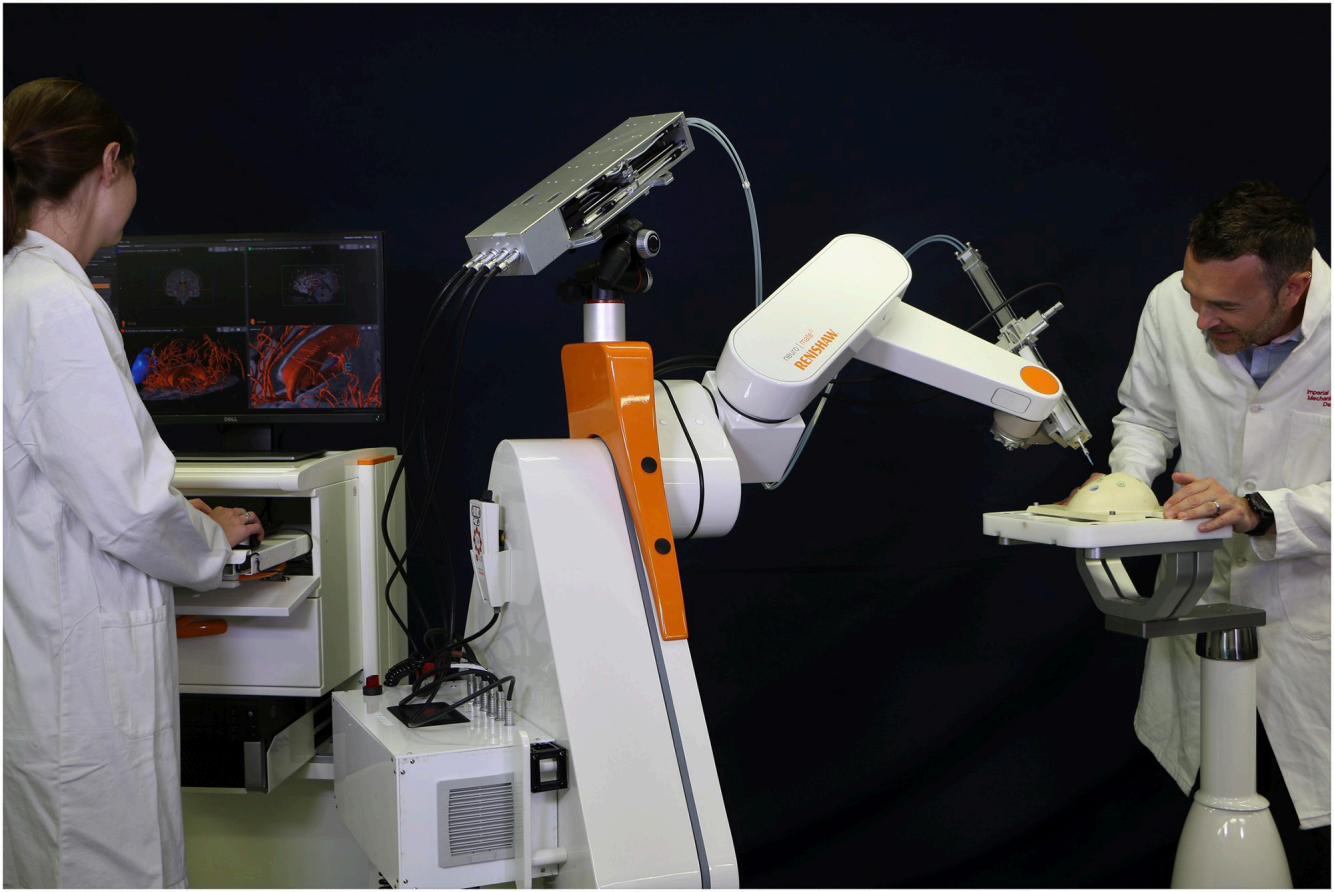
Footprint of the modular robotic platform for precision neurosurgery with a programmable bevel-tip needle. On the right, the modular robotic catheter driver is mounted onto the commercial neurosurgical robot neuromate^®^ (Renishaw plc). On the left the surgeon console with the visual interface.

**Fig 2:**
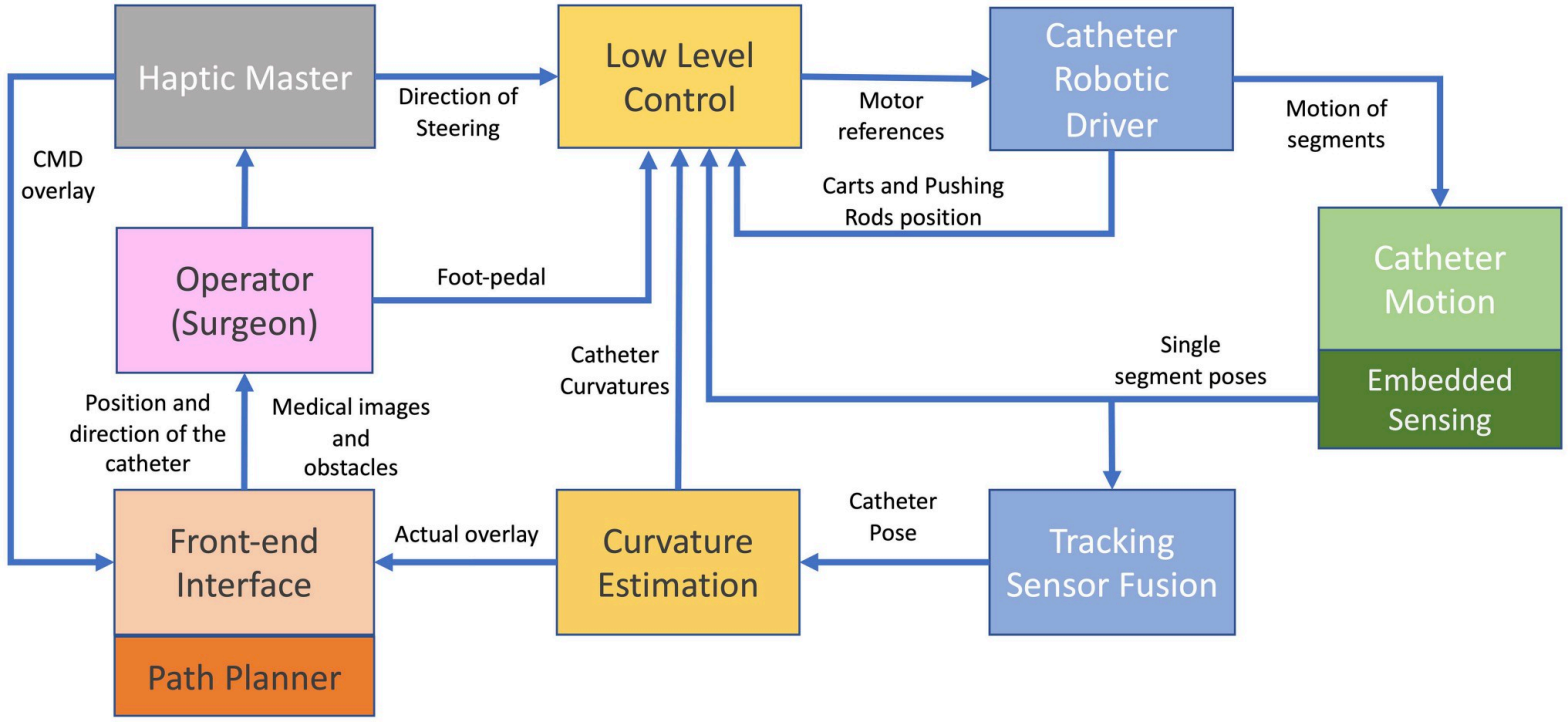
Modules of the architecture of the robotic module ecosystem with human-in-the-loop.

In this paper, we present the first invivo trial of a modular robotic system designed to perform neurosurgical procedures using a programmable bevel-tip needle (PBN). The robotic ecosystem shown in Fig 1, was developed with the aim to provide a clinical tool to assess the potential of Convection Enhanced Delivery (CED) of chemotherapeutics [50] along preferential pathways that align to anisotropic brain structures [51–53]. The paper is structured as follows. Each main module of the robotic system, shown in Fig 2, are detailed by their design, implementation and validation results, as relevant. The Material and Methods section describes the system’s hardware and software components. The hardware subsection describes the programmable bevel tip catheter used in this work, along with a description of the motion system to actuate the insertion (mechatronics actuation box, flexible transmission and user console). Here, a detailed description of the controller developed to compensate for mechanical backlash in the system is also included, followed by a brief description of the sensing scheme utilised to track the catheter shape and tip pose. The software section introduces the user interface, with pre and intra-operative functionalities, including 3D visualisation of the steering path and explicit implementation of a novel algorithm employed to reconstruct point-wise curvatures from a generic path, the application of which extends beyond convection enhanced delivery. Following these, a full system assessment both in *ex vivo* and *in vivo* on an animal model is described, providing insight into the system performance. The paper then concludes with an overview of remaining challenges and future outlook.

## Materials and methods

### Catheter robotic driver

**Fig 3:**
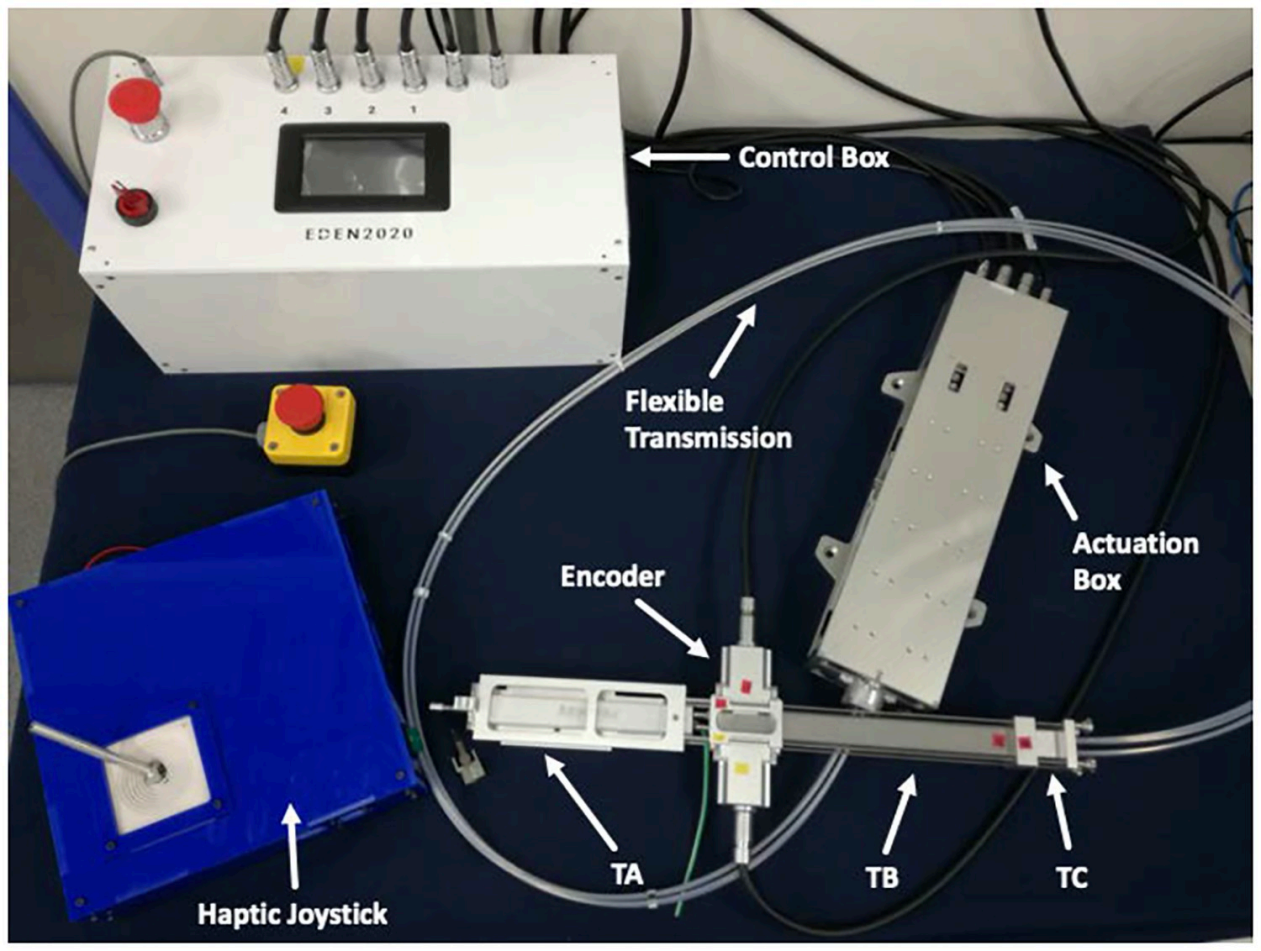
Components of the robotic catheter driver, from top-left clockwise: The control box (in white), the actuation box, the flexible transmission with end-effector (TA-TB-TC), and the haptic joystick.

**Fig 4:**
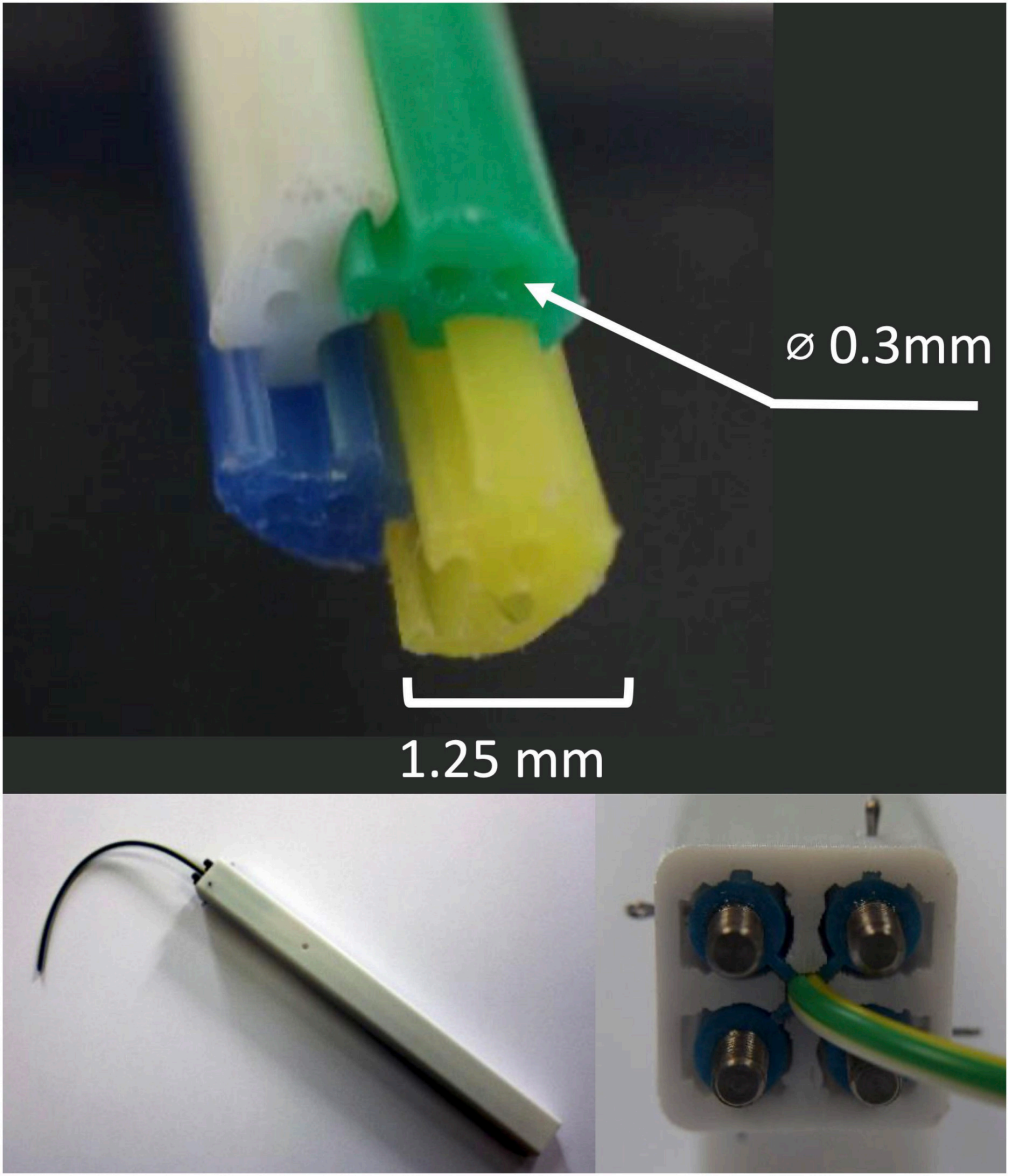
Top: Cross-section of the 4-segment PBN with two 0.3mm diameter working channels per segment. The overall PBN diameter is 2.5mm. Bottom-Left: trocar with embedded medical grade PBN. Bottom-Right: connection mechanism between the PBN wings and the push-rods of TB.

The robotic catheter driver (Fig 3) is formed by four main components: a control box, an actuation box, a flexible transmission and an end-effector (EndE). The control box (the white box in Fig 3) is designed as a stand-alone system and is composed by a Platinum Maestro controller (Elmo Motion Control Ltd.) with four Elmo Twitter motor drivers connected via Ethercat protocol. The actuation box is composed of four custom linear stages (with a linear pitch of 1*mm*/*rev*), each driven by a brushed motor (DC16XS, Maxon Motors AG) featuring embedded co-axial relative encoders with 1024 *pulses/rev*, which enable high precision motion control. A flexible transmission (FT) connects the linear stages to the EndE, as shown in Fig 3-n.5. The actuation box is physically separated from the EndE in order to reduce the mass of the system on the robotic arm end effector, while retaining high repeatability in position. The end-effector is composed of three main subsystems: trocar core A (TA), trocar core B (TB) and the trocar used to connect the flexible transmission (TC), as shown in Fig 3. The TB includes four squared push-rods that provide a sterile/nonsterile interface between the flexible transmission and disposable components. Each push-rod is equipped with a magnetic non-contact linear encoder with a resolution of 0.001*mm* (RLS—Model: RLC2IC). Grouped in pairs, the linear encoder electronics are encapsulated in a sealed, sterilisable metal case. TA is designed to accommodate a disposable medical grade trocar (manufactured in polycarbonate-ISO) that holds the medical-grade PBN, as depicted in Fig 4. Finally, a guillotine system, consisting of two surgical steel scalpel blades, is embedded at the proximal end of TA, which is used to quickly decouple the robot from the catheter during surgery in the event of an emergency).

The PBN used in this work is designed for the specific application of Convection Enhanced Delivery (CED) of chemotherapy drugs directly into brain. It is designed with two lumens per segment (8 lumens in total), one reserved for embedded sensing (e.g. shape sensing or electromagnetic position tracking sensors) and the other to insert an infusion catheter, as show in Fig 4. The PBN is made of nanocoated, medical grade, implantable PVC, with each catheter segment colour coded to facilitate interaction with the clinician, as the intraoperatvie software guides their actions during the infusion process (e.g. screen message: “Insert infusion tube into the BLUE catheter segment”).

### Low level control

The low level control is split into multiple layers: a controller to compensate the flexible transmission backlash, a controller to map the direction of steering imposed by the user onto the catheter in 3D space, and a controller to provide ‘active-constraints’ that help the user to steer towards the target.

#### Flexible transmission backlash compensation

The flexible transmission consists of four nitinol wires longer 1.7m, with 1.6mm in diameter and embedded within a low-friction PTFE tubular casing. The mechanical backlash introduced by the flexible transmission is compensated by a low-level controller composed of a double feedback loop (a magnetic encoder placed at the distal end of the end-effector and an optical encoder placed at the motor side). This is necessary as the mechanical backlash changes as a function of the robotic arm configuration, which in turn depends on the intra-operative setup. If left unchecked, the elastic and frictional effects of the mechanical system could potentially cause an overshoot of the reference target. Hence, a nonlinear shaping function is used to model the velocity of the actuation system in the proximity of the target position. Defining the reference position as *p*_*ref*_ and the actual EndE position as *p*_*a*_, the velocity command *v*_*c*_ for each segment is defined as:

$$\begin{array}{c} v_{c}=\{\begin{array}{cc} v_{r}\cdot \text{tanh}[\frac{p_{ref}-p_{a}}{B}] & p_{ref}-p_{a}>\epsilon \\ 0 & p_{ref}-p_{a}\leq \epsilon \end{array} \end{array}$$

where *B* represents the bandwidth of position by which the controller starts to reduce the velocity and *ϵ* represents the acceptable margin in positional error. *v*_*r*_ defines the average cruise velocity of each catheter segment.

**Fig 5:**
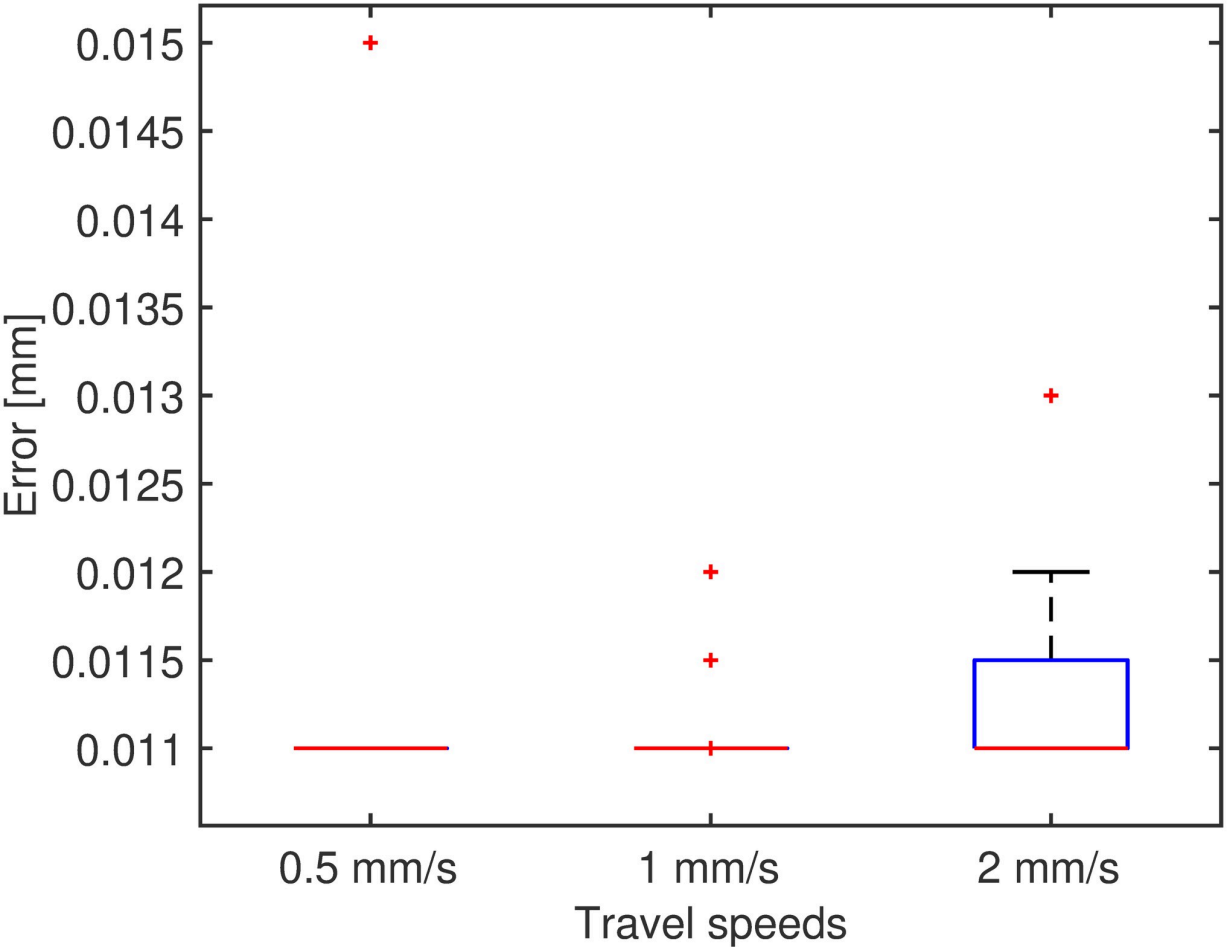
Error in position at the end-effector at three different cruise speeds. Red stars depict outliers.

**Fig 6:**
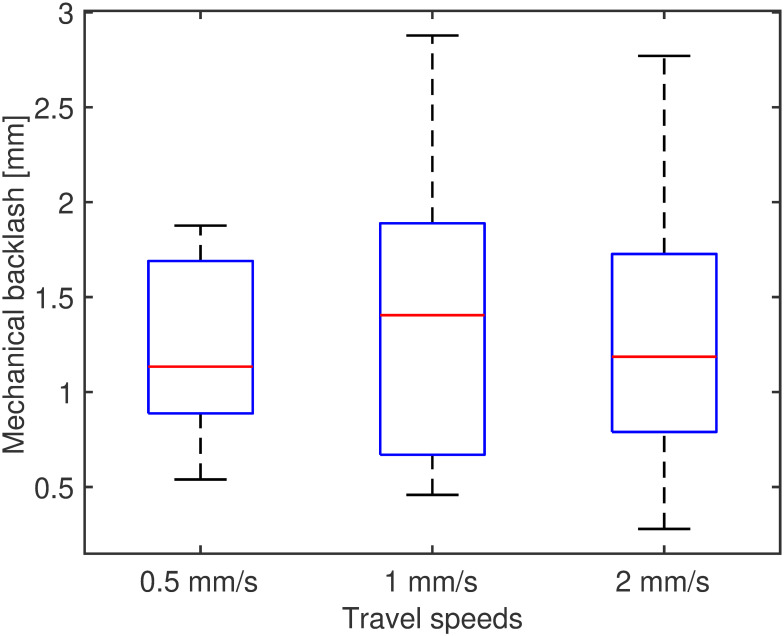
Mechanical backlash in the flexible transmission on the motor side, at three different cruise speeds.

The performance of the system was assessed using a set of trials performed by each actuator at different target positions chosen randomly, respectively in the sequence “10mm, 70mm and 5mm”, with three different velocities, “0.5mms^−1^, 1mms^−1^ and 2mms^−1^”. Three measurements for each case were collected, for a total of 9 trials. For the test, the positional control parameters were set as B = 1mm and *ϵ* = 0.01mm. The results for the positional error are reported in Fig 5. The overall performance of the system, computed as the average positional error in all cases, is 0.011±7.7*e*^−4^mm. The backlash, measured as the distance between the motor and EndE positions at the target end, is reported in Fig 6, with overall results of [0.8011 1.2119 1.7208] mm for the 25^th^, 50^th^ and 75^th^ quantile, respectively. For consistency, the maximum travel velocity for the PBN was set as in previous works [54] to be 1 mms^−1^.

#### Catheter segment mapping

The mapping between the direction of steering imposed by the user and the direction of the catheter has been extensively reported in [55] and here briefly summarised for clarity: The relationship between the steering input (catheter configuration) and the resultant curvature vector may be described by a non-linear function **f**:

$$\begin{array}{c} \kappa =[\begin{array}{c} \kappa_{1}\\ \kappa_{2} \end{array}]=f\left(\Theta \right) \end{array}$$ (1)

where the curvature vector ***κ*** contains the two components of steering corresponding to the two orthogonal axes of the joystick. To steer with a commanded direction and magnitude, a corresponding steering input **Θ** is found. As there may exist more than one catheter configuration to achieve a commanded steering, we formulate the problem as a non-linear programming problem. By optimising a measure of catheter steerability, whilst constraining the commanded curvature with (1), the optimal required steering input is found. In the case that a curvature above the maximum achievable is commanded, the optimisation fails to find a solution and the previous steering input is used.

#### Haptic master

**Fig 7:**
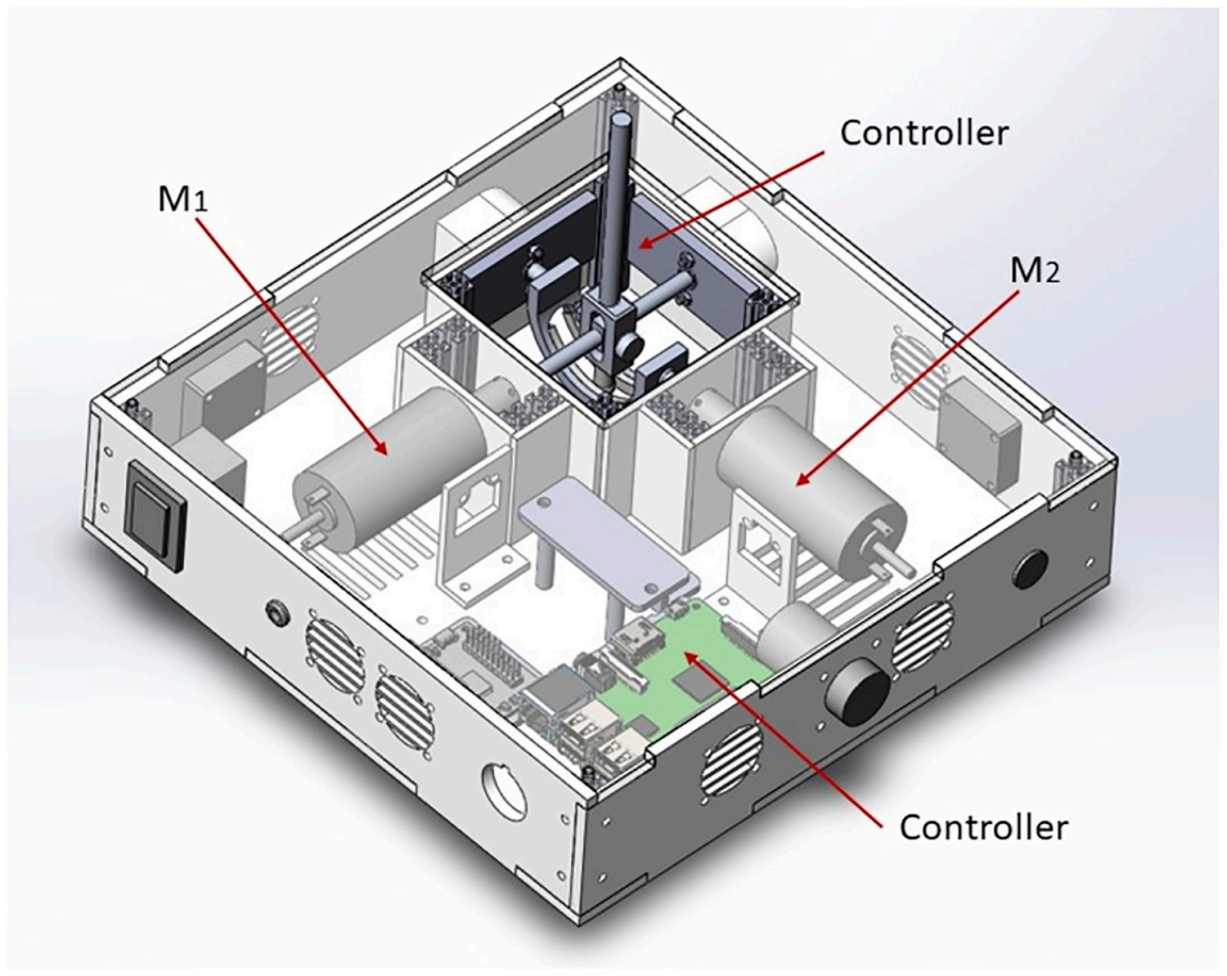
CAD design of the two degree-of-freedom haptic master. The motion of the inner gimbal system is controlled by two independent motors (M1 and M2).

Investigation of different teleoperation masters to control steerable needles were explored in the past [56–58], by using 6DoF haptic systems constrained to 2D or 3D motions. Therefore, we opted for a 2Dof haptic joystick as the master interface for this platform, allowing the user to interact with the robotic catheter system by providing steering commands. The design of the haptic joystick is shown in Fig 7 and it is inspired by the work of [59]. The joystick has a footprint of 250*mm* × 250*mm* × 70*mm*, with a point of grasp for the user 60mm. long. Enclosed in the joystick box are two brushed DC motors (A-max 32 Graphite—20 Watts, Maxon Motor AG) directly linked to the two principal axes of a gimbal mechanism. The gimbal allows a range of motion of ±42*deg* of rotation around each axis by pivoting around the axes intersection point.

A Polytetrafluoroethylene sleeve between the point of contact of the two main axes of the gimbal reduces the contact friction between the moving parts, therefore lowering the transmitting inertia of the motor’s rotors to the user. Two optical encoders (HEDR3600, Avago inc.) mounted directly on the shaft of each motor provide positional feedback to a motor control board (RoboClaw 2x7A, Pololu Corp.) mounted onto a micro-controller (Raspberry Pi3 with a pathched Linux realtime-kernel). Each motor’s parameters were identified empirically and a current closed-loop control was used to provide the desired force to the grasping point. The ideal finger grasping point for the user is designed to be located at 60*mm* from the centre of rotation of the gimbal axes, allowing a maximum rendered force of 1.16*N*. The ergonomics of this gripping strategy mimics the most common pinch grasping use by surgeons during a needle biopsy. To force the user to hold the joystick at the predefined point of grasping, an ergonomic 3D printed plastic handle was mounted over the joystick. A further study to evaluate this new joystick as a master interface with visual and haptic guidance during the control of the insertion of a PBN needle is reported in [60].

### Tracking—Sensor fusion

Several tracking methods can be used to track needles in soft tissue, such as X-Ray fluoroscopy [61], ultrasound (US) [62–64] and electromagnetic (EM) tracking systems [65]. However, imaging methods cannot track the rotation of the needle about its insertion axis (the roll angle) because of its small diameter [5], which also precludes the possibility to accommodate a 6 Degrees of Freedom (DoF) EM sensor. Some authors [62, 66, 67] handled this limitation by considering the roll angle at the needle tip as equal to that measured at the base, assuming an infinite torsional stiffness of the needle, but for soft needle designs, such as the PBN, a different tracking algorithm must be employed. Each segment of the PBN has 2 lumens, as displayed in Fig 4, and to provide the tip-pose reconstruction, one of the lumens can be used to embed sensing such as electromagnetic (EM) trackers, as in [54, 68], or Fiber Bragg Grating (FBG) inscribed fibers, as in [69, 70]. The sensing also provides safety critical information during the catheter insertion, to capture any possible failures of the interlocking mechanism or sliding problems, such as buckling or blockages. As reported in Section, the direction of the steering depends on the non-linear combination of the relative offsets between the PBN segment tips, thus the direction of the leading segment is not sufficient to provide the real direction of steering. Ideally, 6 DoF sensors would be embedded in each segment to estimate the full pose, but to the best knowledge of the authors, no commercial 6 DoF sensors of 0.3mm outer diameter are commercially available.

### Front-end interface

**Fig 8:**
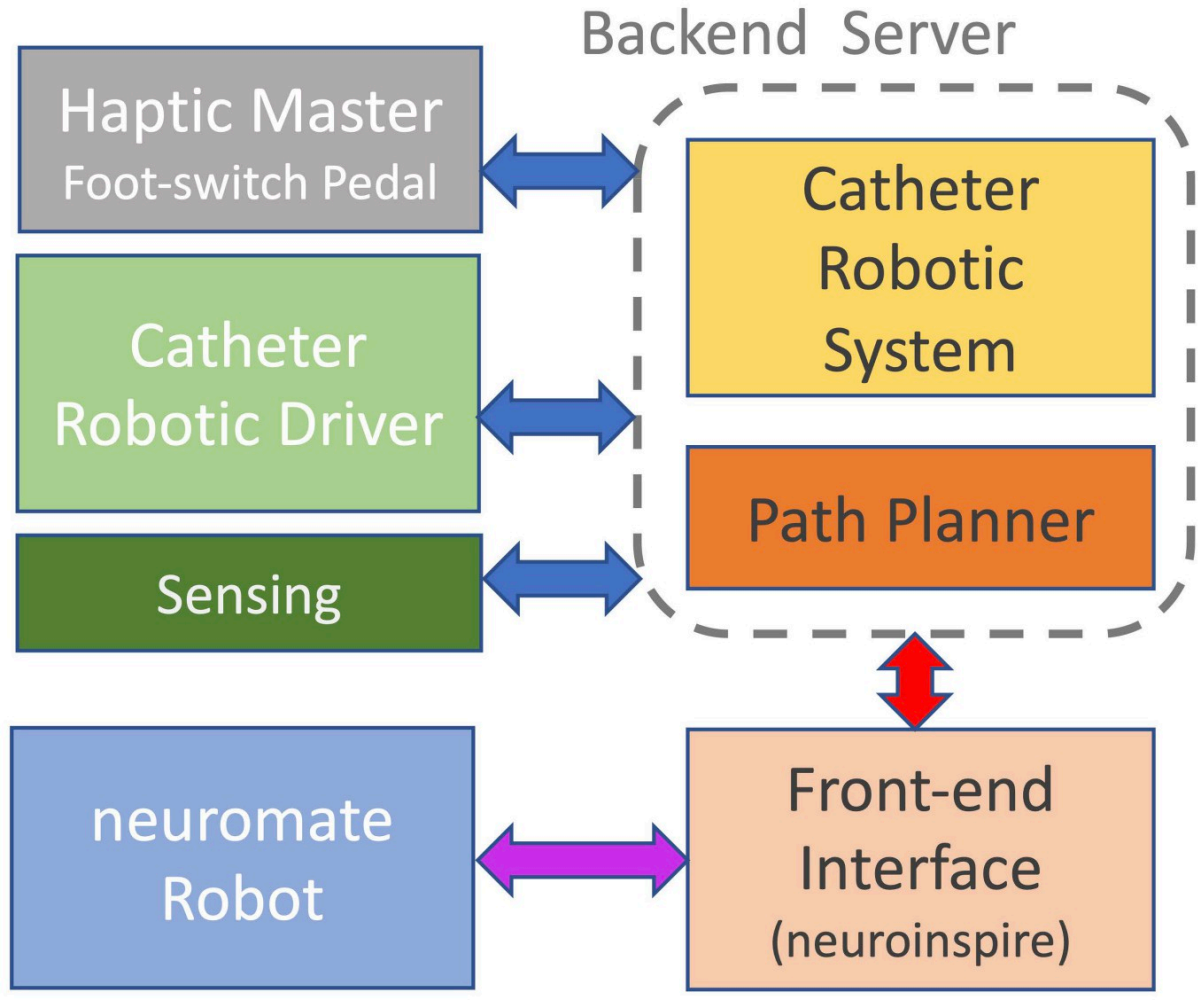
Independent hardware components and communication protocol: In blue, the Robotic Operating System (ROS) protocol. In red, custom proxy communication between the back-end software and the neuroinspire^™^. In purple, proprietary communication protocol between the neuromate^®^ and neuroinspire^™^, as in the commercial system of Renishaw plc.

The software architecture was developed in ROS (version: Kinetic—Linux Ubuntu 16.04) running on multiple machines. Fig 8 provides an overview of all different hardware components communicating using ROS protocols with custom messages (blue arrows), while the interface with the commercial system neuroinspire^™^ was designed with a custom protocol based on ZeroMQ and Google FlatBuffers. The communication and control of the neuromate^®^ robot was kept as the commercial system and proprietary of Renishaw plc.

#### Design

The front-end interface was designed on top of the commercial neurosurgical planning and intra-operative software neuroinspire^™^ (Renishaw plc, UK). The standard functionalities of the software, such as the preoperative registration of MRI and CT images, displays of medical image datasets using three conventional orthogonal views, and renders of the three dimensional volume on a fourth view, were kept as per the original software. The new visual interface incorporates the rendering of tractography computed from Diffusion Tensor Imaging (DTI) and segmentation import function (e.g. vessels from angiography and other structures) both for pre- and intra-operative planning. With the term segmentation, we refer to 3D models generated as the output of different processes where the clinician identifies anatomical structures of interest (from medical image intensities, functional areas and/or an anatomical atlas). The segmentation output is encoded in the STL file format, which can subsequently be imported into the planner, such that it can be used for the computation of the optimal insertion path. Custom views to support planning (drug selection, target selection, planner options, burr-hole port placement, path selection and visualisation, etc.), devices initialisation and intra-operative navigation were added.

#### Preoperative plan

**Fig 9:**
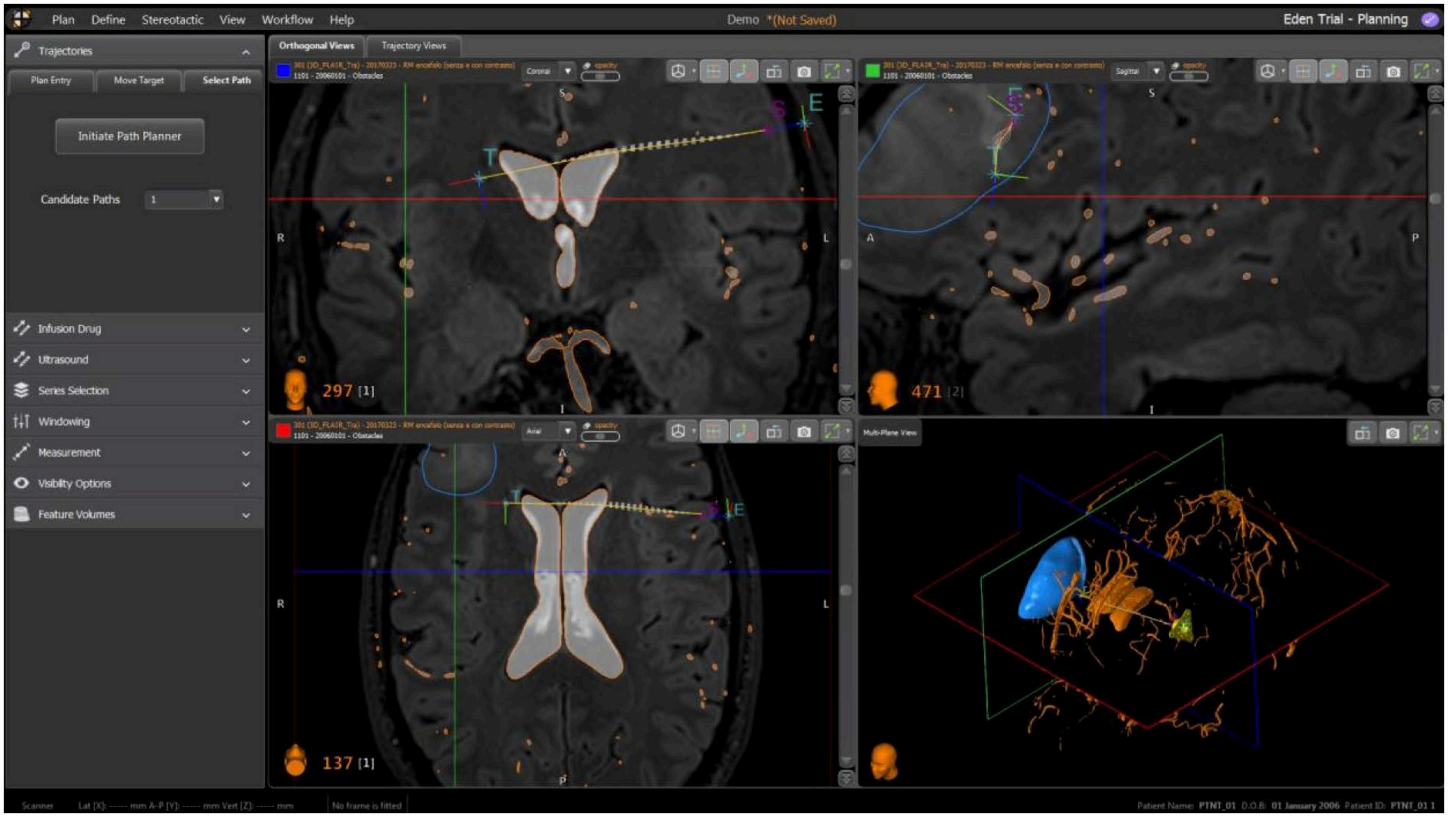
Preoperative planner: Rendering of the entry point in the skull with burr-hole port positioning, feasible paths to reach the predefined target, obstacles and target rendered as 3D meshes.

The path that the surgeon should follow is generated pre-operatively by the planner, as in [71], which avoids all obstacles within a safe radius, as shown in Fig 9.

The workflow of the pre-operative planner is as follows:

1. The user imports the imaging datasets (CT, MR, etc.)
2. The user follows imaging registration steps in the software
3. The user imports the segmentation of anatomical structures needed by the back-end to compute the target pose (e.g. white matter, grey matter, ventricles, target area)
4. The user imports segmentations of all the obstacles the planner should consider
5. The user chooses the infusion drug type and volume
6. The back-end software computes and displays the infusion catheter target point and direction, and the drug flow rate according to [72]
7. The back-end software computes a set of candidate entry points, that are perpendicular to the skull’s surface, with a 15% degrees tolerance. The user selects their preferred option. There are options to freely modify or manually redefine the entry point if required. A model of the burr-hole port is displayed to facilitate the positioning according to user preference
8. The back-end software computes all of the paths that are feasible by the catheter and are free from obstacles. They are ranked according to a specific metric that aims to minimise the insertion length and the number of bends to limit tissue damage and increase precision. The front-end displays a maximum of 10 candidate paths according to the metric
9. The user selects their preferred path among the proposed candidate paths

The software was developed with the option to add intra-operative ultrasound (US) image guidance. During the pre-operative planning phase, further steps are dedicated to the optimal position of the US probe.

#### Intra-operative navigation

The workflow for the intra-operative module summarised in the following section, while here we describe the intra-operative guidance display used during navigation, rendered on the 4^*th*^ view of the front-end interface.

**Fig 10:**
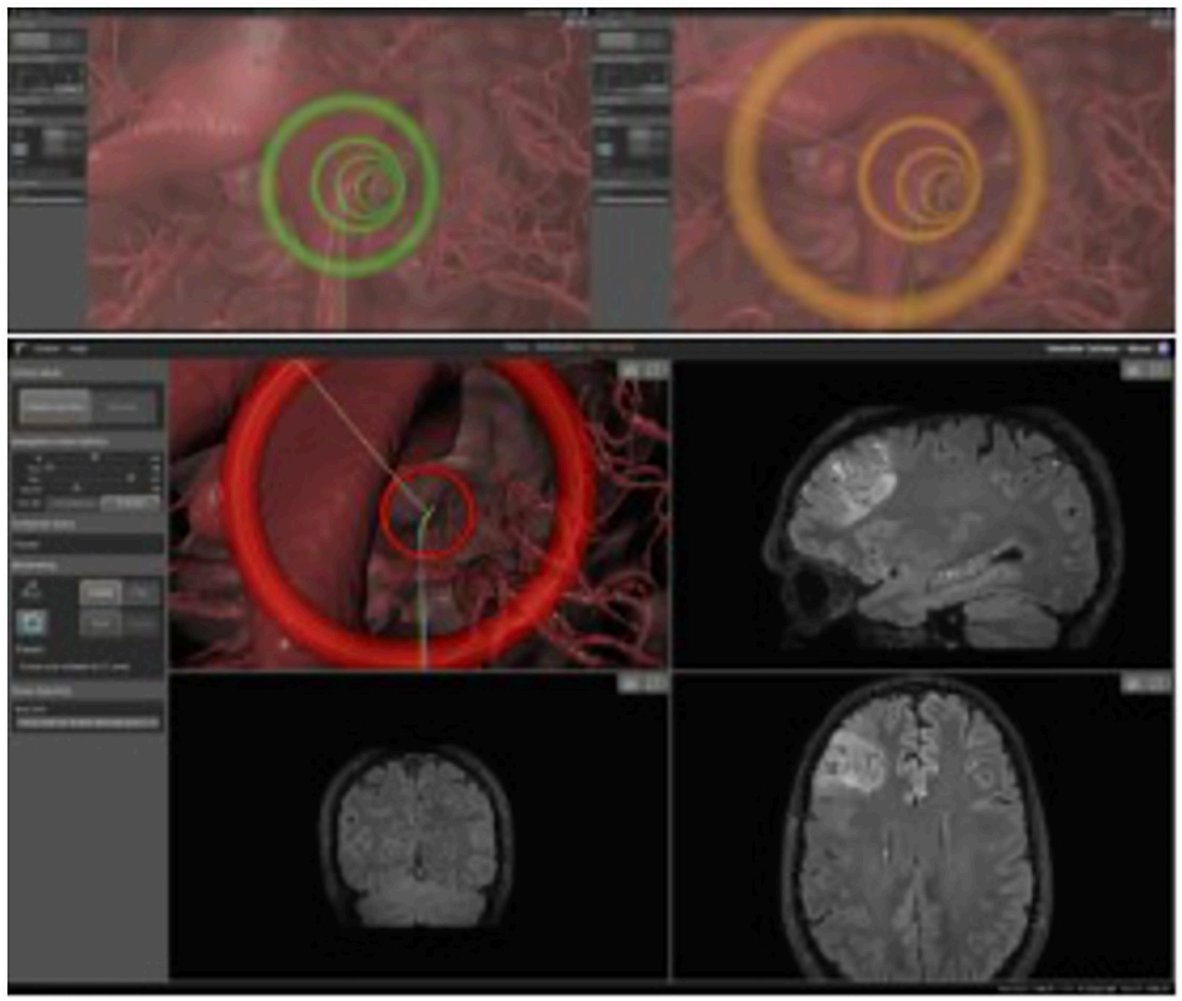
Catheter view mode when error is low (top left), getting higher (top right) and high, hence triggering path re-planning (bottom).

**Fig 11:**
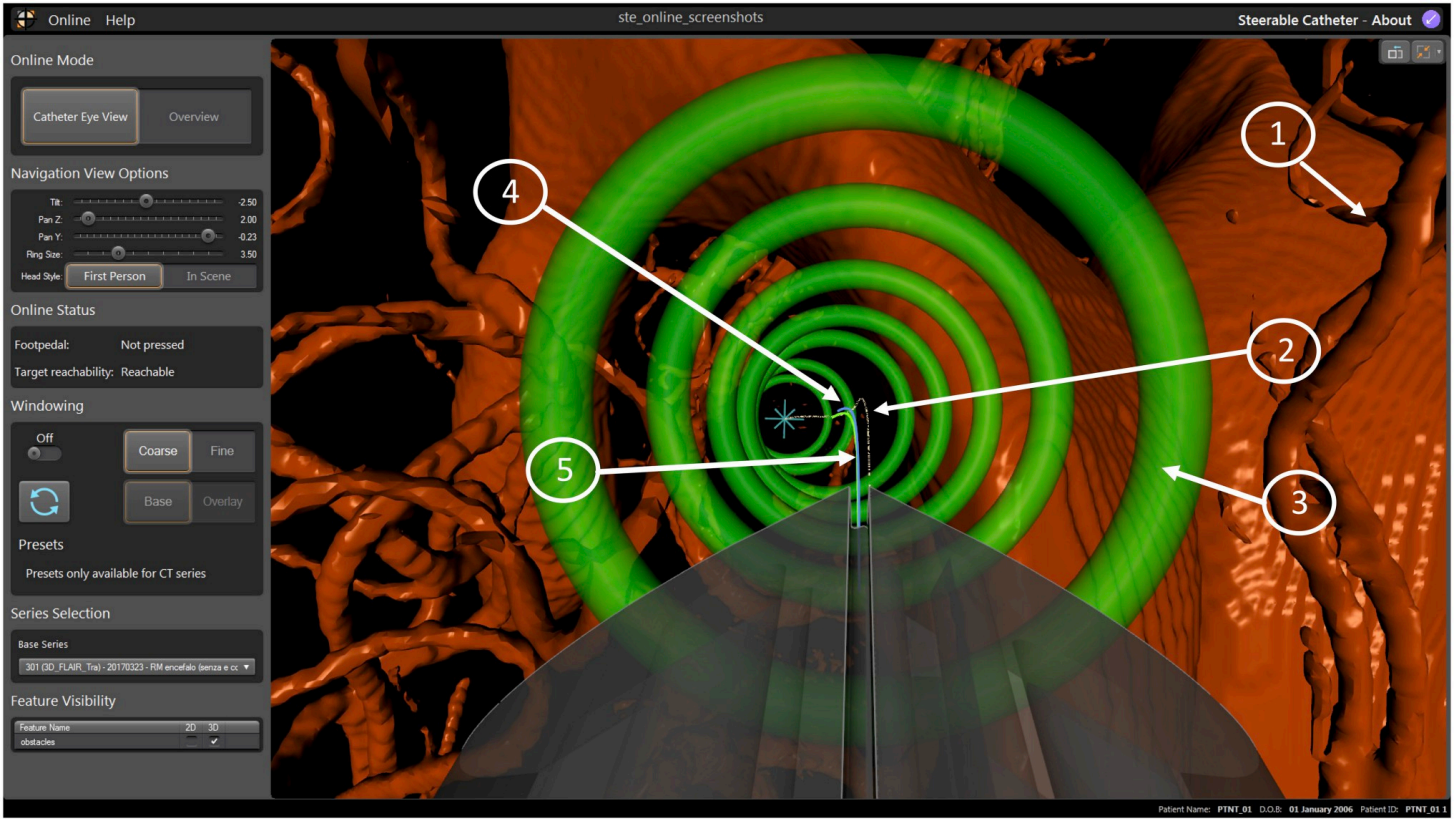
Visual interface with catheter view mode: 1) Obstacles meshes, 2) reference path, 3) waypoints, 4) actual overlay, 5) commanded overlay.

This fourth view renders the anatomical features of the brain, as well as the catheter and cues for intuitive steering. Specifically, the preoperative MRI images are segmented in order to create 3D obstacles maps. With the additional use of the US intra-operative imaging, these 3D obstacles are deformed in real-time according to measurements of the US [73]. The surgeon can then steer through this map in order to reach the desired target, shown in Fig 11. The intra-operative navigation starts with the visualisation of the pre-operative path selected by the surgeon at point (9) of the pre-operative workflow described earlier. If, during catheter insertion, the surgeon deviates from the path by a predefined magnitude, then the system will immediately re-plan a new feasible path [74] to the target, if one exists. The intra-operative re-planner computes obstacle-free paths considering the current catheter tip pose and any change in the obstacles configuration, while remaining as close as possible to the previous path. If no such path exists, a message is displayed to inform the surgeon that, due to the current surgical status and considering the kinematic constraints of the catheter, the target is not reachable with the planned pose. The surgeon is then able to decide whether to stop the operation or continue with the catheter insertion, with the caveat that the target will be reached sub-optimally. Following a detailed clinical consultation with stakeholders, the last generated path is always displayed on screen (see Fig 10) to provide the surgeon with a visual reference in the latter case.

**Fig 12:**
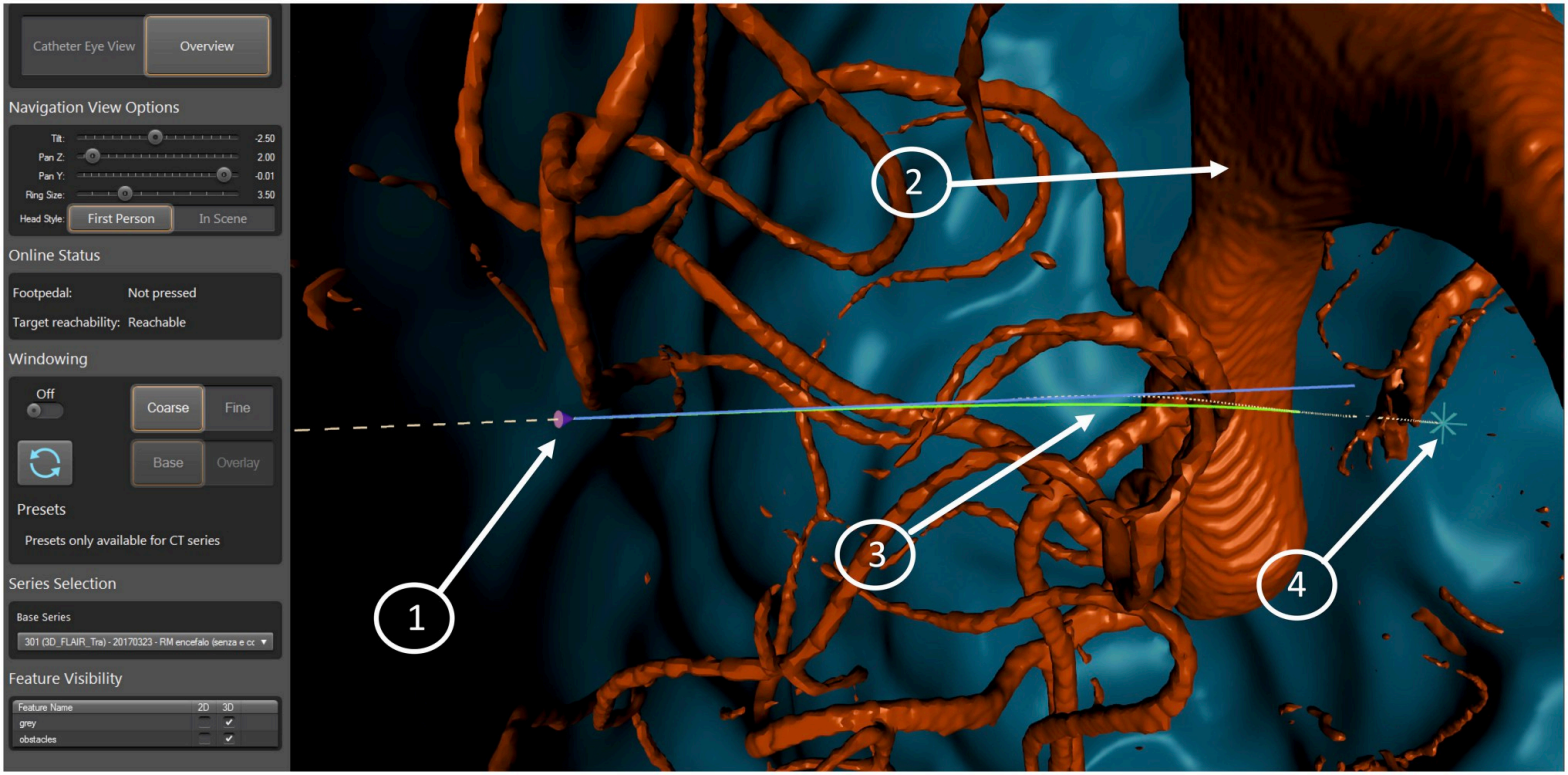
Visual interface with overview mode: 1) Current pose of the catheter, 2) obstacles, 3) optimal path, 4) target position.

The path that the surgeon should follow, as well as the current configuration of the tip of the catheter, can be visually depicted to the surgeon in multiple ways. In this design, the navigation window shows a 2D render of a 3D environment, where the surgeon has a first person viewpoint when navigating the catheter (called the Catheter View Mode, see Fig 11), though they can also choose to stop steering and look from a third person view (called the Overview Mode, see Fig 12) in order to overview the full trajectory.

This design was chosen based on feedback from an advisory group of neurosurgeons, as it was hypothesized that it would be more intuitive for a surgeon to navigate in first person view, though the third person overview was also needed in order to show a similar viewpoint as normally seen when looking at standard MRI volumes. For the same reason, the selected image (MR, CT) is cut along the three orthogonal planes and displayed in the other three windows. The cutting point is defined by the current position of the catheter tip, as detected by the sensing. The catheter tip and the outline of the segmented obstacles and target area are overlayed on the slices to give additional feedback to the surgeon. Each of the four individual windows can be maximized as needed. The navigation window in Catheter View Mode (Fig 11) has the following visual components:

1. Selected segmentations representing the obstacle (e.g. anatomical arterial tree, ventricles, no-go areas)
2. Optimal path depicted as a white line
3. Waypoints represented as rings, where the centre of the ring lies on the optimal path. The color of the rings is a function of the error distance between the catheter tip pose to the path (“green>orange>red” mean “close>further>far”)
4. Blue ray represents the “Actual Overlay”
5. Green ray represents the “Commanded Overlay”

The navigation window in Overview Mode (Fig 12) has the following visual components:

1. Current pose of the catheter tip shown as a purple cone
2. Selected segmentations representing obstacles
3. Optimal path (dashed white line) and overlay rays
4. Target position represented as a star

The “Actual Overlay” represents the current path the catheter is following based on a local reconstruction of the curvatures in the Parallel Transport frame. This is further described in the next section. The “Commanded Overlay” represents the path the catheter should follow based on the configuration of the joystick and inverse kinematics of the catheter. The colour of the ring represents a metric for the error indicating how far the catheter is from the path, and fuses the magnitude of position and orientation error into a value between 0 (directly on the path) and 1 (far from the path, in which case a path re-planning event is triggered). Quantitative and qualitative results from a user studies trial for the visual interface are reported in previous work [60].

### Curvature estimation

Considering the complexity and low dynamics of the system, the time-lag between a joystick command and the visual steering of the catheter could considerably effect the reaction of the user in following the predefined path. To help the user, the ‘Actual Overlay’ path is represented in the front-end interface to provide information regarding the real-time direction of the catheter according to the actual configuration of the catheter.

To reconstruct the “Actual Overlay” path, it is necessary to reconstruct the pose of the catheter and to infer the curvature that the tip is following. Therefore, it is necessary to reconstruct the local curvature, which as in [75, 76], we have defined by the Parallel Transport frame. The estimation of the curvature of the Parallel Transport frame has been developed considering a differential geometry perspective in *SE*(3), where the Lie group G is viewed as a differentiable Riemannian manifold, and the Lie algebra is the tangent space at the identity of the Lie group. This method can be used with any nonholonomic system defined as a particle-mass moving in space, and thus not just limited to programmable bevel tip needle designs.

We define the two maps of the Lie group G, which maps locally a point on the geodesic on G through **Π**: the exponential map expm_**Π**_ and the logarithm map logm_**Π**_. The map forms the tangent space $T_{\Pi }G$ to the Lie group G, and is defined as:

$$\begin{array}{c} {\text{expm}}_{\Pi }:T_{\Pi }G\to G \end{array}$$ (2)

while the inverse mapping is defined as:

$$\begin{array}{c} {\text{logm}}_{\Pi }:G\to T_{\Pi }G \end{array}$$ (3)

Specifically, we assume that the catheter is moving over a normalised geodesic on Riemannian manifold along a curve *γ* defined as:

$$\begin{array}{c} \gamma \;:\;s\to (\begin{array}{cc} R(s) & P(s)\\ 0 & 1 \end{array}) \end{array}$$

parametrised in *s*. The derivative at the identify element is:

$$\begin{array}{c} \Pi =(\begin{array}{cc} \Omega & V\\ 0 & 0 \end{array}) \end{array}$$ (4)

with **Ω** representing the rotational velocity while **V** represents the linear velocity. According to equation map (2), we have:

$$\begin{array}{c} \gamma (s)=\text{expm}(s\Pi ) \end{array}$$

**Algorithm 1** Parallel Transport Frame Curvatures

1: **procedure**:

2:  Input: position of points $p_{t_{0}}$, $p_{t_{1}}$, $p_{t_{2}}$ ∈ℜ_3×1_

3:  Output: Curvatures *K*_1_, *K*_2_

4:  $t\leftarrow \frac{p_{t_{1}}-p_{t_{0}}}{∥p_{t_{1}}-p_{t_{0}}∥}$     ⊳ Tangent vector

5:  **n**_**1**_ ← [−**t**_3_**t**_1_0]     ⊳ Normal vector

6:  **n**_**2**_ ← **t** × **n**_**1**_     ⊳ Binormal Vector

7:  **T**_**B1**_ ← [**n**_**1**_ **n**_**2**_ **t**]     ⊳ Tangential Bundle $p_{t_{0}}p_{t_{1}}$

8:  $t_{2}\leftarrow \frac{p_{t_{2}}-p_{t_{1}}}{∥p_{t_{2}}-p_{t_{1}}∥}$

9:  **b** ← **t**_**2**_ × **t**

10:  costh ← **t**_**2**_ ⋅ **t**

11:  Δ ← ‖**b**‖

12:  $B\leftarrow [\begin{array}{ccc} 0 & -b_{3} & b_{2}\\ b_{3} & 0 & -b_{1}\\ -b_{2} & b_{1} & 0 \end{array}]$     ⊳ **b** = [*b*_1_*b*_2_*b*_3_]

13:  **if** Δ > 0 **then**

14:   $R_{M}\leftarrow \left[1+\frac{\left(1-\text{costh}\right)}{\Delta^{2}}\right]\cdot B$

15:  **else**

16:   $R_{M}\leftarrow I_{3}$

17:  **end if**

18:  **n**_**12**_ ← **R**_**M**_ ⋅ **n**_**1**_

19:  **n**_**22**_ ← **t**_**2**_ × **n**_**12**_

20:  **T**_**B2**_ ← [**n**_**12**_**n**_**22**_**t**_**2**_]

21:  $\Pi \leftarrow \frac{\text{logm}[T_{B2}^{⊺}T_{B1}]}{∥p_{t_{2}}-p_{t_{1}}∥}$

22:  $\Omega \to [\begin{array}{ccc} 0 & 0 & -k_{2}\\ 0 & 0 & k_{1}\\ -k_{1} & k_{2} & 0 \end{array}]$     ⊳ From Eq 4

23: **return** *k*_1_, *k*_2_

24: **end procedure**

The estimation of the curvature is obtained with Algorithm n.1 with a Z-forward configuration, where the following assumptions are made: the catheter tip is moving along a curve *γ* over a riemannian geodesic in *SO*(3), and there are at least three consecutive time points of the tip position such that the travel speed between two consecutive points is assumed constant and unitary over the arclength of the curve, to satisfy the conditions of Hopf-Rinow Theorem. The Algorithm n.1 uses the property of Eq 3 to calculate the curvatures (*k*_1_ and *k*_2_) defined in the Parallel Transport Frame [77]. In the case of multiple points on the curve, Algorithm n.1 is iterated from point 8 to 23 to reconstruct the frame. For the full mathematical demonstration and definition of Eqs 2 and 3, we refer to [78].

### Full system *in vitro* validation

**Fig 13:**
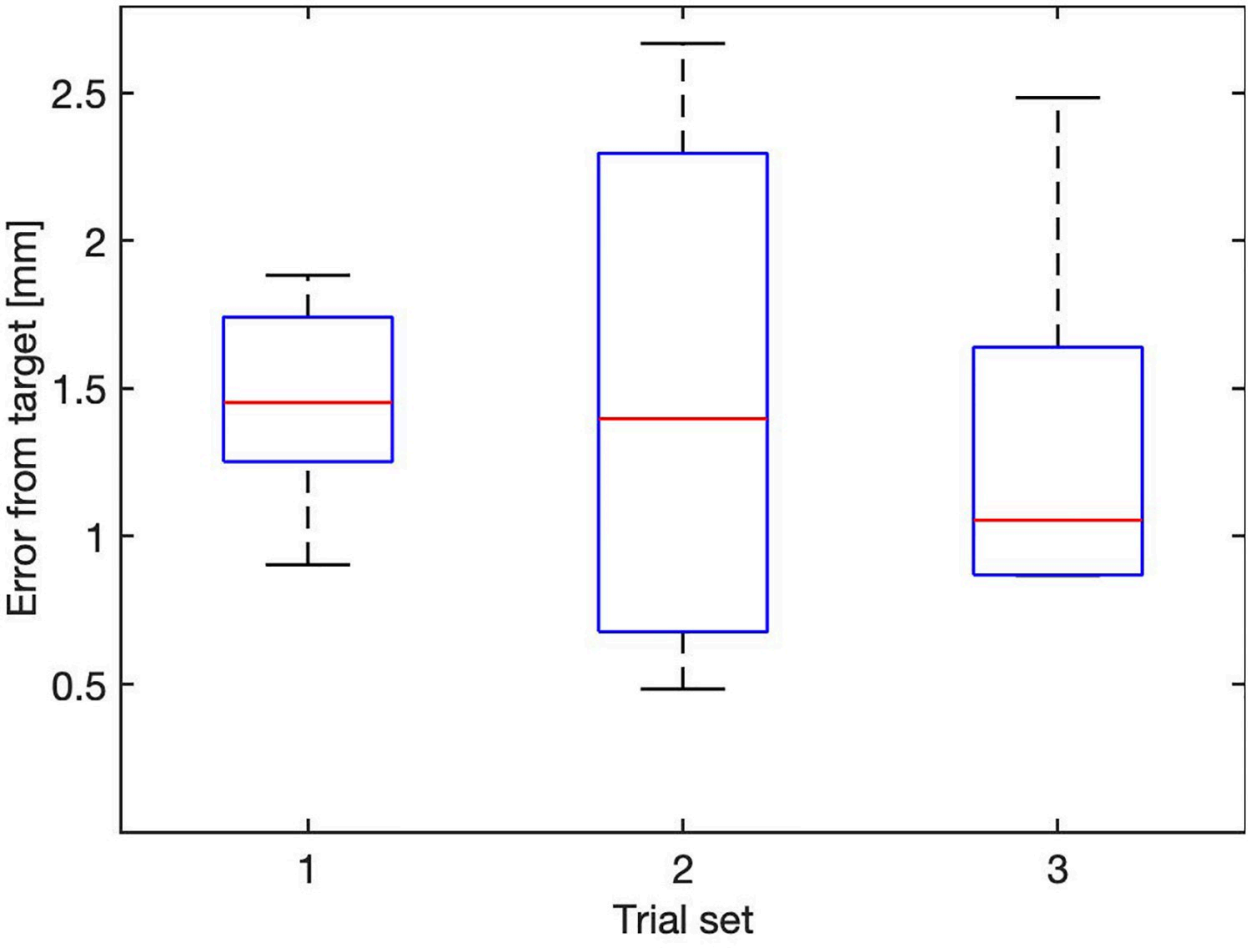
Errors recorded during *in vitro* validation of the system, computed as the euclidean distance between the tip of the catheter and target position.

To validate the system performances with all components integrated, a set of *in vitro* trials were performed in 6% by weight bovine gelatin (Chef William Powdered Gelatine) with a setup as in [68]. The reference paths were defined using the pre-operative path planner presented in [74] on an anonymous patient data-set [79]. An expert user performed 5 trials on 3 different paths, for a total of 15 insertions, with an average insertion length of 110mm. To assess the usability of the system, all insertions were executed with visual feedback active. An investigation of the performance of fully automated steering is presented in [74], where a path re-planner was used to generate steering inputs to replace the user. In this context, a threshold for activation of the path-planner was arbitrarily set at 2mm error from the reference path. The position error achieved during the trials with the expert user was calculated as the average euclidean distance between the tip of the catheter and the target, with resulting values of: [0.871.361.88] mm respectively for the 25^th^, 50^th^ and 75^th^ percentiles, and reported per trial set in Fig 13

### Ethics statement

The individual pictured in Fig 1 has provided written informed consent (as outlined in PLOS consent form) to publish their image alongside the manuscript

#### Ethics protocol

All animals (ovine model, *ovis aries*—average weight of 70*kg*, females, average one year old) were treated in accordance with the European Communities Council directive (2010/63/EU), to the laws and regulations on animal welfare enclosed in D.L.G.S. 26/2014. Ethical approval for this study was obtained by Milan University Animal Welfare Organization (OPBA) and the Italian Health Department with authorization n° 635/2017-PR of August 7, 2017.

#### Anaesthesia protocol

Animals were anesthetized via the intravenous administration of Diazepam 0.25*mg*/*Kg* and Ketamine 5*mg*/*Kg*, intubated and then maintained under general anesthesia with isoflurane 2% and oxygen 2*L*/*min*. Two peripheral venous accesses in right and left auricular veins were set for each sheep and urinary catheterization performed. A Ruminal probe was placed to to prevent tympanism.

#### Euthanasia protocol

Overdose of intravenous potassium chloride under anesthesia

### *Ex vivo* surgical workflow assessment

**Fig 14:**
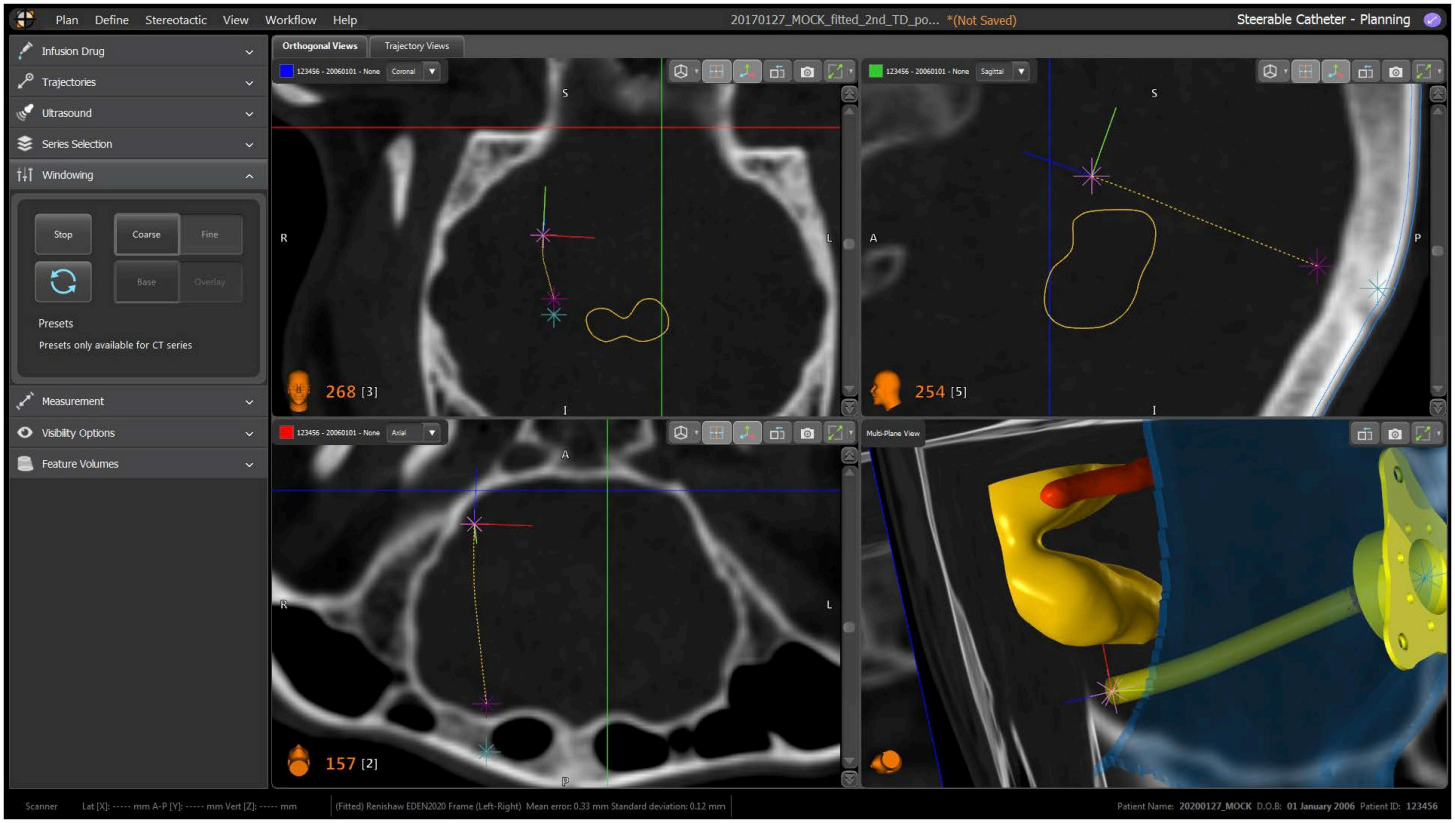
Software interface for pre-operative planning: Conventional image view (in clockwise order from the top left: Axial, sagittal, 3D rendering and coronal). The “T” marker represents the target, the orange meshes represent the obstacles (ventricles and veins), the blue mesh is the skull surface. In yellow are feasible entry points.

**Fig 15:**
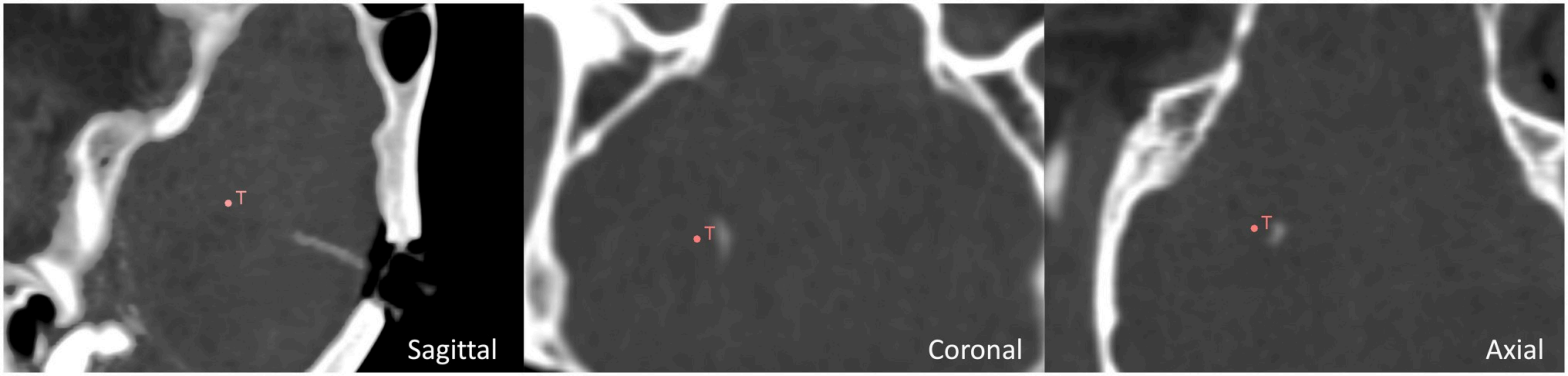
Ex vivo post-operative CT image. *T* defines the target point, while the white shadow is the catheter.

A validation of the full system in an *ex vivo* clinical setting was performed to assess the surgical workflow and the system in light of the *invivo trial*. The embedded sensors used were FBG fibers as in [69]. The fibers were inserted into one of the two working channel of each segment, and secured at the tail end of the catheter to avoid relative motion during the insertion process. The procedure is divided in eight major steps, as follow:

1. The sheep is located on a spinal stretcher (acrylonitrile butadiene styrene, ABS stretcher, Millenia, Ferno) and is secured in a prone position on a vacuum mattress with extended legs, via two straps.
2. The Head Frame System described in [80], is placed onto the stretcher and secured using a custom fixture system
3. The animal head is fixed into the Head Frame System described in [80]
4. Acquisition of the pre-operative CT imaging sequence (pre-operative CT—GE Healthcare CT system, 16 slices helical scan). The imaging sequences were acquired with a standard display field of view (DFOV), 512x512 matrix, 0, 625mm. slice thickness, 120 kilovolt (KV), 220 milliampere (mA), 0, 562: 1 pitch and 1/*s* tube rotation. The images were collected using a soft tissue algorithm)
5. Acquisition of a pre-operative MRI (Siemens 1.5T, 3DT1 Fast-Field-Echo, DTI, TOF). DTI imaging is loaded from the dataset of a previous study [81]
6. The surgeon performs the pre-operative planning sequence following steps in section *Preoperative plan*, shown in Fig 14
7. The surgeon performs the intra-operative planning and navigation sequence in section
8. A second CT imaging sequence is recorded (post-operative CT) to assess the positioning of the catheter, as in Fig 15
9. Acquisition of a post-operative MRI (Siemens 1.5T, 3DT1 Fast-Field-Echo, DTI, TOF)
10. Insertion of the infusion catheter to perform infusion of Gadolinium

### Intraoperative planning sequence

**Fig 16:**
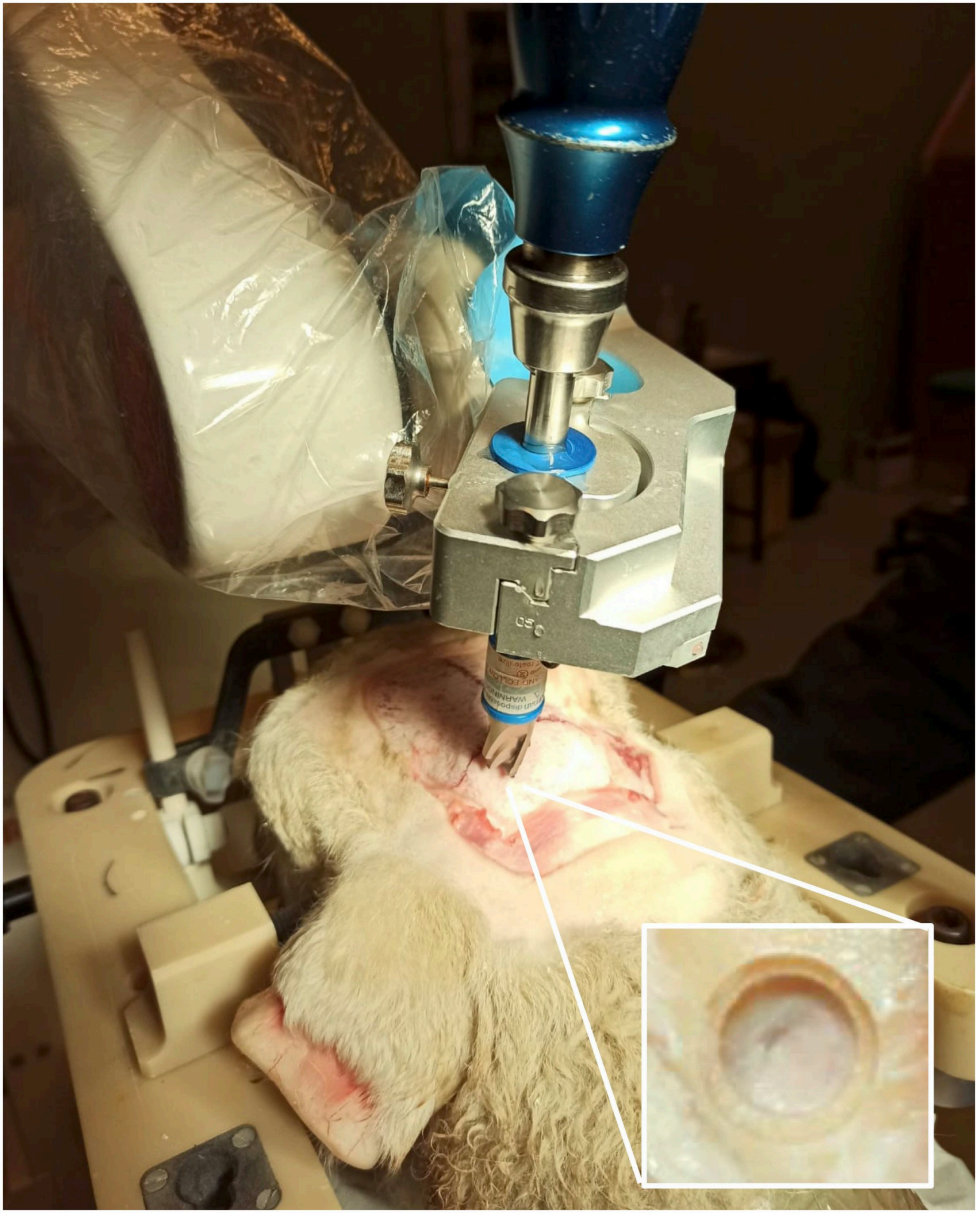
Surgical perforator mounted at the end-effector of the neuromate^®^. Zoomed window: profile left on the skull by the surgical perforator.

**Fig 17:**
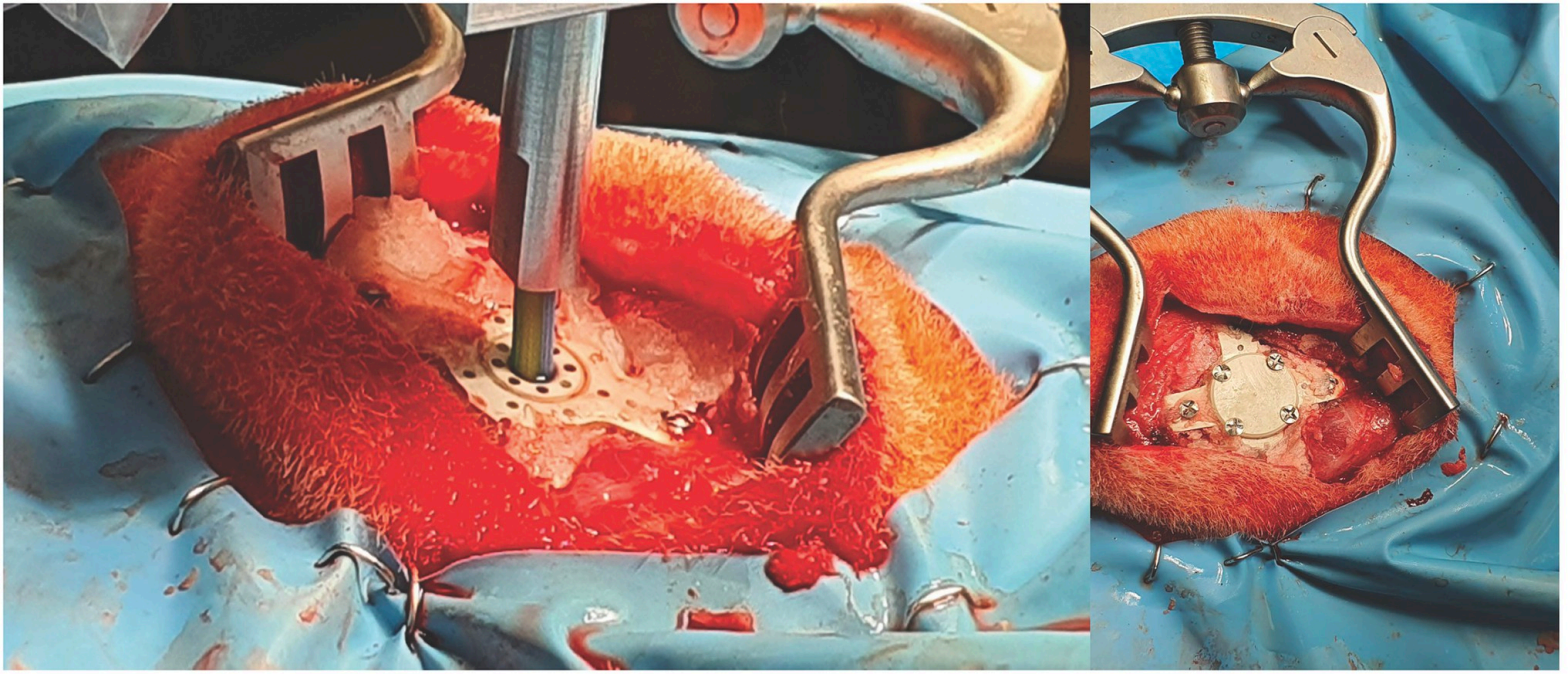
(on the left) catheter insertion on the dura matter. (on the right) keyhole port is sealed after the inserted catheter is detached by the robotic system.

The following procedure (Step 7) is carried out once pre-operative planning is complete:

7a) The surgical preoperative plan is loaded into the front-end interface
7b) A custom drill holder for the neuromate^®^ is mounted and, once the robot in position, a keyhole on the skull is made, as in Fig 16 (the tools to perform the keyhole are: J&J Anspach^®^ EMAX^®^ 2 Plus with gearbox reduction and a cranial perforator model Codman 14mm).
7c) The surgeon creates a small incision on the dura
7d) A custom burr-hole port is placed into the keyhole and secured using titanium screws
7e) The neuromate^®^ end-effector is moved back to the parking position and the drill holder is replaced with the robotic catheter driver end-effector
7f) The neuromate^®^ end-effector is positioned over the burr-hole port and the catheter is automatically driven to the entry-point position in the brain, as shown in Fig 17
7g) The front-end interface is switched to catheter-view mode, as in Fig 10, and control of the insertion moves to the surgeon
7h) The surgeon starts the insertion by pressing a foot-pedal. The PBN is inserted at a constant speed of 1mm/s and the direction is controlled by the haptic-joystick operated by the surgeon. If the foot-pedal is released, the catheter stops. If the foot-pedal is pressed once more, the insertion resumes.
7i) Once the target is reached, the surgeon secures the catheter on the skull by using a hidden locking mechanism within the burr-hole port
7j) The sensing embedded in the catheter is removed by pulling the fibers from the tail of the catheter
7k) The catheter is cut flush to the top surface of the burr-hole port and the robot end-effector is moved to the parking position

**Fig 18:**
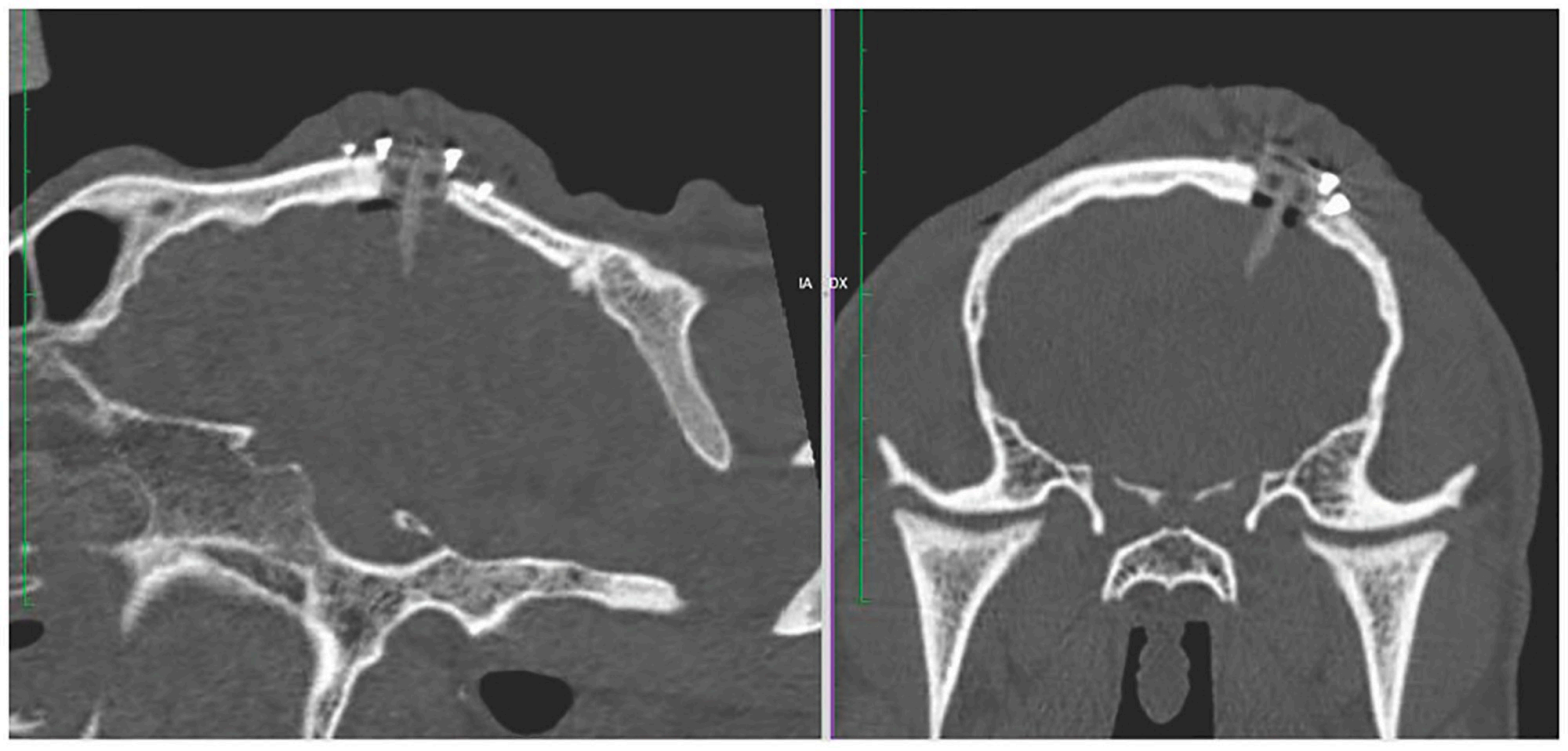
CT scan after the surgery.

At the final step of the procedure, the front-end interface provides a message reporting which coloured segment of the PBN is suitable for the infusion, as only the system can compute the segment which is closest to the target. The infusion of drugs is delivered with an additional catheter having an outer diameter of 0.3mm and inner diameter of 0.1 mm, which is inserted in the desired segment’s working channel. This latter part of the protocol, as well as the infusion performance, are out of scope for this work. Following the surgery, a CT image is collected to define the final position of the catheter with respect to the surgical plan (Step 8). As shown in Fig 15, which shows an image of the post-operative CT acquisition for the *ex vivo* trial, the catheter has reached the target with an euclidean error in position of 1.42mm.

## Results

### First *In-vivo* evaluation

The *in-vivo* assessment of this platform technology aimed to establish the effectiveness of the surgical workflow for keyhole neurosurgery and the feasibility and safety of PBN delivery and implantation (5 days) within the brain, for eventual application to CED. In order to carry out this trial safely during the COVID-19 pandemic, a reduced clinical workflow was favoured, with a skeleton surgical team on site and minimum transfers of equipment and sheep in and outside of the operating theatre. Notably, due to the more stringent health and safety requirements of our chosen veterinary facility, the imaging facilities of which were off site, MR scanning was obviated by employing an ovine statistical atlas [81, 82] produced by the investigator team.

**Fig 19:**
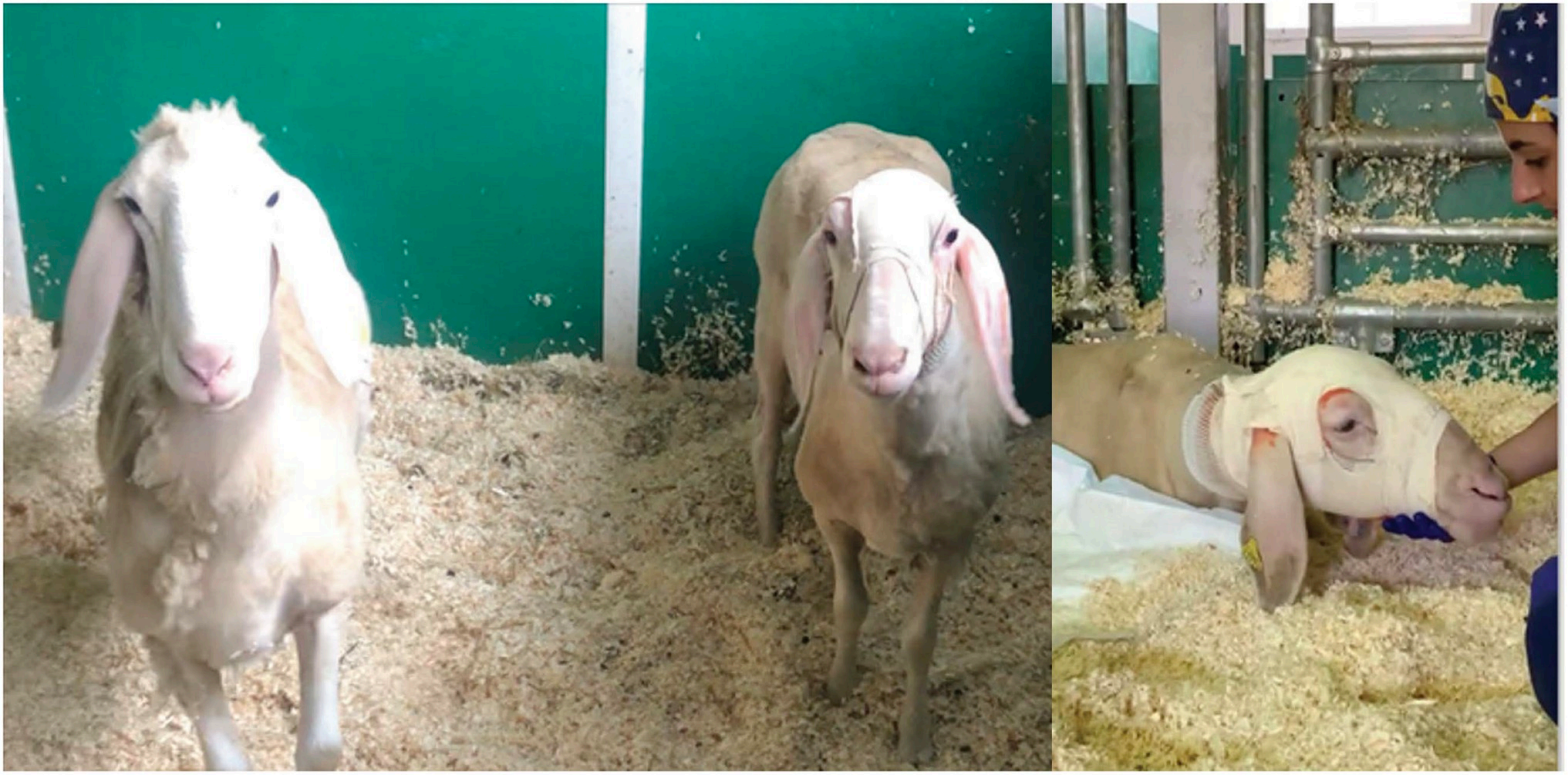
Animal management after surgery.

**Fig 20:**
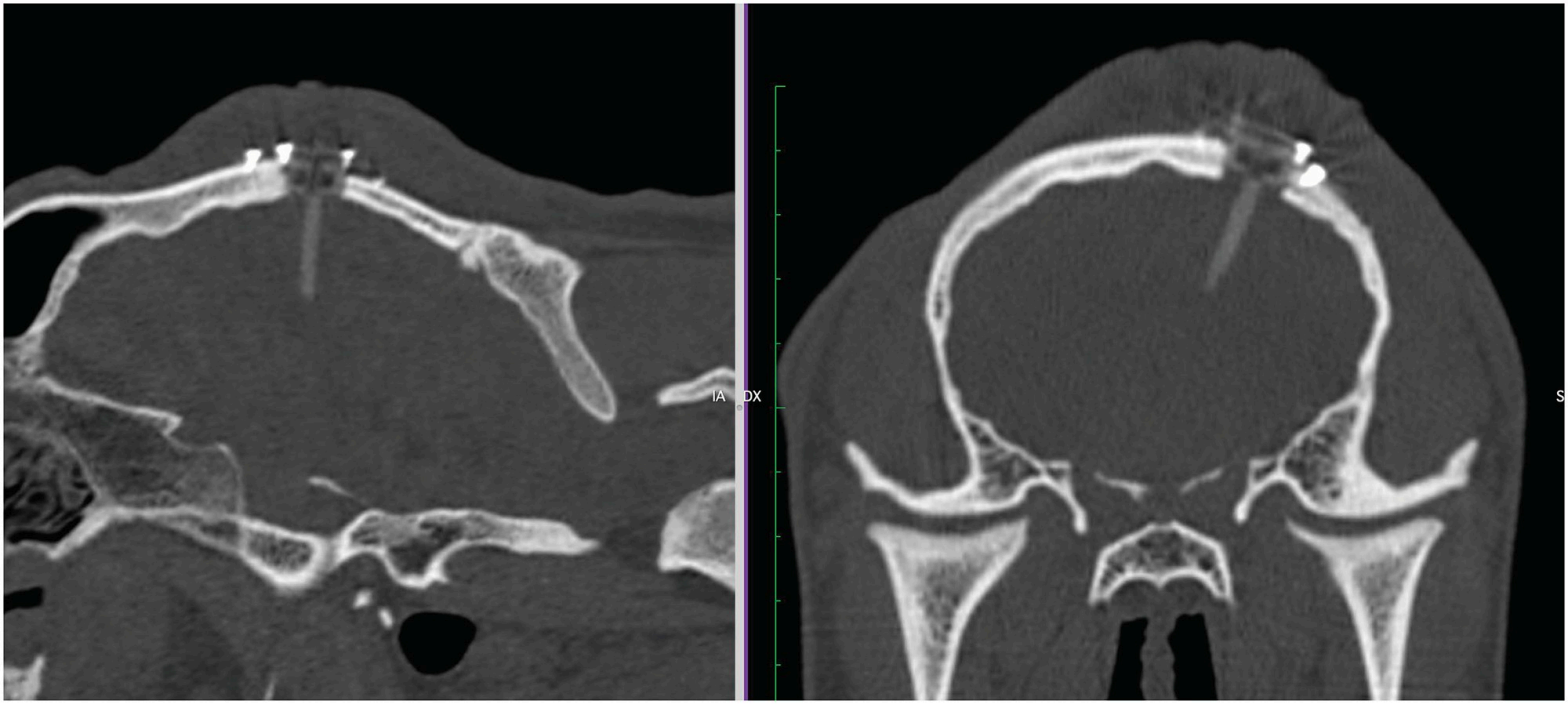
CT scan before animal sacrifice at day 5: CT slice after surgery at day 0 (left); same CT slice at five days (right).

**Fig 21:**
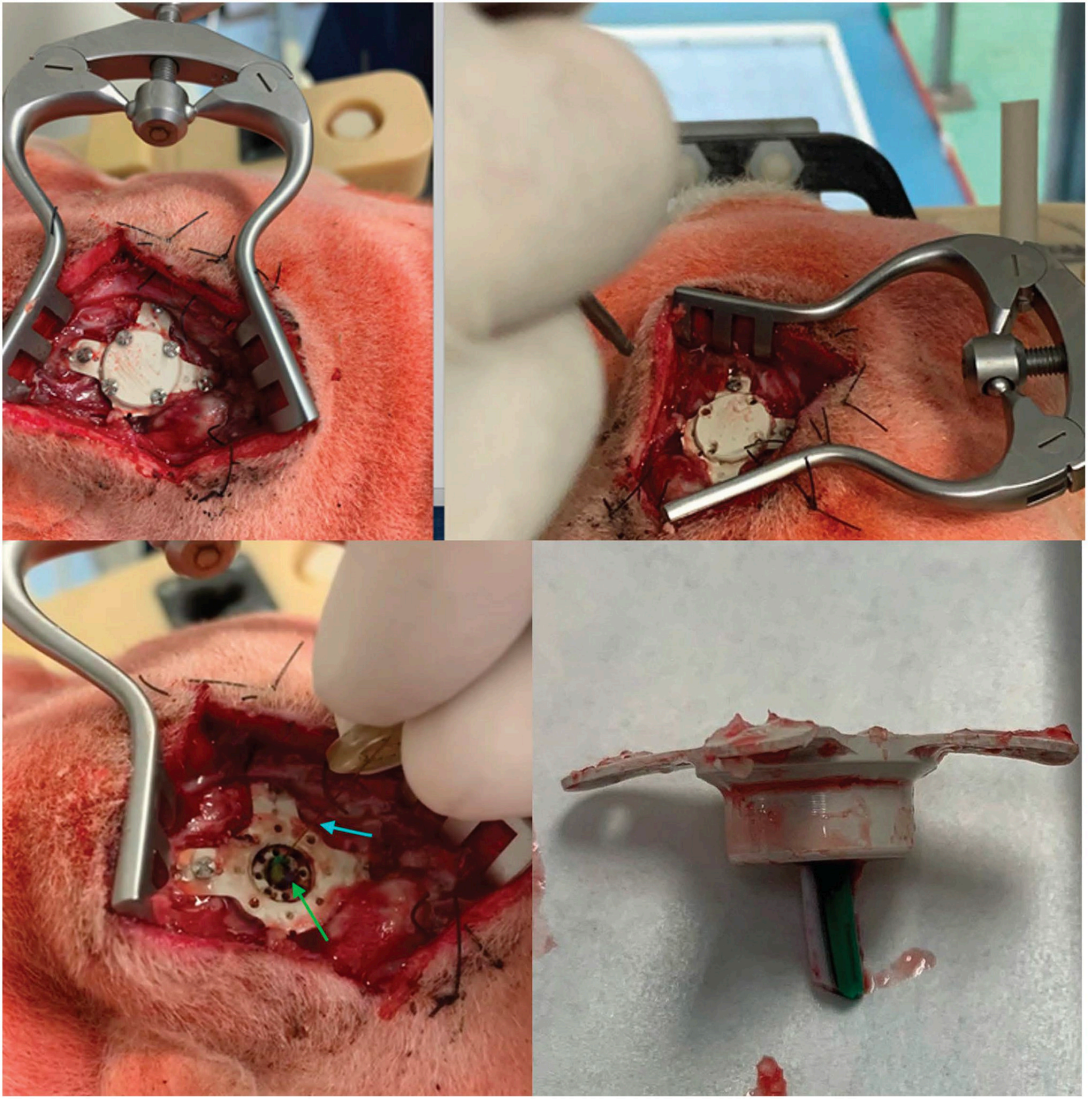
Opening of the surgical site at day 5 (top). Insertion of the infusion catheter to assess working channel function (bottom left). Port and catheter extracted post-mortem (bottom right).

According to the surgical workflow, a CT scan of the surgical site was taken after surgery, as illustrated in Fig 18. The image shows the needle successfully implanted, with a total length of insertion of 12.33*mm* from the surface of the brain to the tip of the needle (22.24*mm* if computed as the length from the external surface of the port to the tip) and with an error of 0.9*mm* between preoperative and achieved target. After surgery, the animal was awoken and brought into the housing area where it underwent a period of clinical evaluation lasting at least 2 hours, as shown in Fig 19. Clinical data was recorded using a dedicate ethogram, where parameters are assessed at 12-hour intervals for five days. No abnormal or out-of-normal parameters were noted throughout the observation period. According to the ethical protocol, the animal was sacrificed at day five and a CT of the surgical site was taken before assessing any occlusion of the catheter working channels following the 5-day implantation period. Fig 20 shows the CT image of the catheter before sacrifice, while Fig 21 shows the surgical site before the assessment of possible working channels occlusions. The burr-hole port and surrounding tissue were also visually inspected, with no visible sign of infection, confirmed by blood and cerebral fluid sample analyses.

## Discussion and conclusion

In this work, we have presented the details and first *in vivo* assessment of a modular robotic ecosystem for precision neurosurgery, which employs a programmable bevel-tip steerable needle (PBN). The design has taken into account the functional requirements of the operating theatre (OR), including ergonomics and sterility. The front-end interface, as well as the workflow, have been developed in collaboration with a clinical team within the EDEN2020 European consortium, to provide a system that can be deployed in the OR, with a streamlined clinical workflow. The overall performance in positioning the catheter is comparable to results of analogous systems [83], both in bench-testing and during *ex vivo* assessment.

Though this first live trial confirmed that the system is functional and catheter implantation can be performed safely, additional investigations and further *in vivo* trials are planned to improve the efficiency and efficacy of catheter placement and to assess performance during Convection Enhanced Delivery (CED). Future works will also include the introduction of additional sensing, such as intraoperative ultrasound imaging, to provide measurements of tissue deformation during surgery. The additional sensing will also be fused with the embedded sensing within the catheter segments to provide improved localisation of the catheter tip. This integration will allow the system to compensate for target motion due to tissue deformation, which can affect the final positioning accuracy of the catheter at the target and along the path.

## References

[1] Dupont PE, Nelson BJ, Goldfarb M, Hannaford B, Menciassi A, O’Malley MK, et al. A decade retrospective of medical robotics research from 2010 to 2020. Science Robotics. 2021;6(60):eabi8017. doi: 10.1126/scirobotics.abi8017 34757801 PMC8890492

[2] Westebring van der Putten EP, Goossens RHM, Jakimowicz JJ, Dankelman J. Haptics in minimally invasive surgery—a review. Minimally Invasive Therapy & Allied Technologies. 2008;17(1):3–16. doi: 10.1080/13645700701820242 18270873

[3] Van de Berg NJ, van Gerwen DJ, Dankelman J, van den Dobbelsteen JJ. Design Choices in Needle Steering; A Review. IEEE/ASME Transactions on Mechatronics. 2015;20(5):2172–2183. doi: 10.1109/TMECH.2014.2365999

[4] Abolhassani N, Patel R, Moallem M. Needle insertion into soft tissue: A survey. Medical Engineering and Physics. 2007;29(4):413–431. doi: 10.1016/j.medengphy.2006.07.003 16938481

[5] Cowan NJ, Goldberg K, Chirikjian GS, Fichtinger G, Alterovitz R, Reed KB, et al. Robotic needle steering: Design, modeling, planning, and image guidance. In: Surgical Robotics: Systems Applications and Visions. Boston, MA: Springer US; 2011. p. 557–582.

[6] Misra S, Reed KB, Schafer BW, Ramesh KT, Okamura AM. Mechanics of Flexible Needles Robotically Steered Through Soft Tissue. The International Journal of Robotic Research. 2010;29(13):1640–1660. doi: 10.1177/0278364910369714 21170164 PMC3002232

[7] Majewicz A, Marra SP, van Vledder MG, Lin M, Choti MA, Song DY, et al. Behavior of tip-steerable needles in ex vivo and in vivo tissue. IEEE transactions on bio-medical engineering. 2012;59(10):2705–2715. doi: 10.1109/TBME.2012.2204749 22711767 PMC3448818

[8] Rossa C, Tavakoli M. Issues in closed-loop needle steering. Control Engineering Practice. 2017;62:55–69. doi: 10.1016/j.conengprac.2017.03.004

[9] Mignon P, Poignet P, Troccaz J. Automatic Robotic Steering of Flexible Needles from 3D Ultrasound Images in Phantoms and Ex Vivo Biological Tissue. Annals of Biomedical Engineering. 2018;46(9):1385–1396. doi: 10.1007/s10439-018-2061-3 29845413

[10] Cotler MJ, Rousseau EB, Ramadi KB, Fang J, Graybiel AM, Langer R, et al. Steerable Microinvasive Probes for Localized Drug Delivery to Deep Tissue. Small. 2019;15(37):1901459. doi: 10.1002/smll.201901459 31183933

[11] Webster RJ, Jones Ba. Design and Kinematic Modeling of Constant Curvature Continuum Robots: A Review. The International Journal of Robotics Research. 2010;29(13):1661–1683. doi: 10.1177/0278364910368147

[12] Dupont PE, Lock J, Itkowitz B, Butler E. Design and control of concentric-tube robots. IEEE Transactions on Robotics. 2010;26(2):209–225. doi: 10.1109/TRO.2009.2035740 21258648 PMC3022350

[13] Gilbert HB, Rucker DC, Webster III RJ. Concentric Tube Robots: The State of the Art and Future Directions. In: Inaba M, Corke P, editors. Robotics Research—The 16th International Symposium ISRR, 16-19 December 2013, Singapore. vol. 114 of Springer Tracts in Advanced Robotics. Springer; 2013. p. 253–269.

[14] Glozman D, Shoham M. Image-guided robotic flexible needle steering. IEEE Transactions on Robotics. 2007;23(3):459–467. doi: 10.1109/TRO.2007.898972

[15] Neubach Z, Shoham M. Ultrasound-Guided Robot for Flexible Needle Steering. IEEE Transactions on Biomedical Engineering. 2010;57(4):799–805. doi: 10.1109/TBME.2009.2030169 19709957

[16] Fichtinger G, Fiene JP, Kennedy CW, Kronreif G, Iordachita I, Song DY, et al. Robotic assistance for ultrasound-guided prostate brachytherapy. Medical Image Analysis. 2008;12(5):535–545. doi: 10.1016/j.media.2008.06.002 18650122 PMC4724791

[17] Kallem V, Cowan NJ. Image Guidance of Flexible Tip-Steerable Needles. IEEE Transactions on Robotics. 2009;25(1):191–196. doi: 10.1109/TRO.2008.2010357 20431694 PMC2860577

[18] Engh JA, Minhas DS, Kondziolka D, Riviere CN. Percutaneous Intracerebral Navigation by Duty-Cycled Spinning of Flexible Bevel-Tipped Needles. Neurosurgery. 2010;67(4):1117–1123. doi: 10.1227/NEU.0b013e3181ec1551 20881576

[19] Rucker DC, Das J, Gilbert HB, Swaney PJ, Miga MI, Sarkar N, et al. Sliding Mode Control of Steerable Needles. IEEE Transactions on Robotics. 2013;29(5):1289–1299. doi: 10.1109/TRO.2013.2271098 25400527 PMC4231884

[20] Krieger A, Song S, Cho NB, Iordachita II, Guion P, Fichtinger G, et al. Development and Evaluation of an Actuated MRI-Compatible Robotic System for MRI-Guided Prostate Intervention. IEEE/ASME Transactions on Mechatronics. 2013;18(1):273–284. doi: 10.1109/TMECH.2011.2163523 PMC3544166 23326181

[21] Fallahi B, Rossa C, Sloboda RS, Usmani N, Tavakoli M. Sliding-Based Switching Control for Image-Guided Needle Steering in Soft Tissue. IEEE Robotics and Automation Letters. 2016;1(2):860–867. doi: 10.1109/LRA.2016.2528293

[22] DiMaio SP, Salcudean SE. Interactive Simulation of Needle Insertion Models. IEEE Transactions on Biomedical Engineering. 2005;52(7):1167–1179. doi: 10.1109/TBME.2005.847548 16041980

[23] Roesthuis RJ, van de Berg NJ, van den Dobbelsteen JJ, Misra S. Modeling and steering of a novel actuated-tip needle through a soft-tissue simulant using Fiber Bragg Grating sensors. In: 2015 IEEE International Conference on Robotics and Automation (ICRA). IEEE; 2015. p. 2283–2289.

[24] Ryu SC, Quek ZF, Koh JS, Renaud P, Black RJ, Moslehi B, et al. Design of an Optically Controlled MR-Compatible Active Needle. IEEE Transactions on Robotics. 2015;31(1):1–11. doi: 10.1109/TRO.2014.2367351 26512231 PMC4620588

[25] Seong Young Ko, F Rodriguez y Baena. Toward a Miniaturized Needle Steering System With Path Planning for Obstacle Avoidance. IEEE Transactions on Biomedical Engineering. 2013;60(4):910–917. doi: 10.1109/TBME.2012.2227741 23193445

[26] Scali M, Kreeft D, Breedveld P, Dodou D. Design and evaluation of a wasp-inspired steerable needle. vol. 10162. International Society for Optics and Photonics; 2017. p. 1016207.

[27] Ayvali E, Liang CP, Ho M, Chen Y, Desai JP. Towards a discretely actuated steerable cannula for diagnostic and therapeutic procedures. The International Journal of Robotics Research. 2012;31(5):588–603. doi: 10.1177/0278364912442429 22639482 PMC3359092

[28] Konh B, Sasaki D, Podder TK, Ashrafiuon H. 3D Manipulation of an Active Steerable Needle via Actuation of Multiple SMA Wires. Robotica. 2020;38(3):410–426. doi: 10.1017/S0263574719000705

[29] Ilami M, Ahmed RJ, Petras A, Beigzadeh B, Marvi H. Magnetic Needle Steering in Soft Phantom Tissue. Scientific Reports. 2020;10(1):2500. doi: 10.1038/s41598-020-59275-x 32051477 PMC7016187

[30] Faria C, Erlhagen W, De Momi E, Ferrigno G, Bicho E. Review of Robotic Technology for Stereotactic Neurosurgery. IEEE reviews in biomedical engineering. 2015;. doi: 10.1109/RBME.2015.2428305 25955851

[31] Smith JA, Jivraj J, Wong R, Yang V. 30 Years of Neurosurgical Robots: Review and Trends for Manipulators and Associated Navigational Systems. Annals of Biomedical Engineering. 2016;44(4):836–846. doi: 10.1007/s10439-015-1475-4 26467553

[32] Fomenko A, Serletis D. Robotic Stereotaxy in Cranial Neurosurgery: A Qualitative Systematic Review. Neurosurgery. 2017;83(4):642–650. doi: 10.1093/neuros/nyx576 29253265

[33] Wang MY, Goto T, Tessitore E, Veeravagu A. Introduction. Robotics in neurosurgery. Neurosurgical Focus FOC. 2017;42(5):E1. doi: 10.3171/2017.2.FOCUS1783 28463607

[34] Kwoh YS, Hou J, Jonckheere EA, Hayati S. A robot with improved absolute positioning accuracy for CT guided stereotactic brain surgery. IEEE Trans Biomed Eng. 1988;35(2):153–160. doi: 10.1109/10.1354 3280462

[35] Drake JM, Joy M, Goldenberg A, Kreindler D. Computer- and robot-assisted resection of thalamic astrocytomas in children. Neurosurgery. 1991;29(1):27–33. 1870684 10.1097/00006123-199107000-00005

[36] Li QH, Zamorano L, Pandya A, Perez R, Gong J, Diaz F. The application accuracy of the NeuroMate robot—A quantitative comparison with frameless and frame-based surgical localization systems. Computer Aided Surgery. 2002;7(2):90–98. doi: 10.3109/10929080209146020 12112718

[37] von Langsdorff D, Paquis P, Fontaine D. In vivo measurement of the frame-based application accuracy of the Neuromate neurosurgical robot. Journal of Neurosurgery JNS. 2015;122(1):191–194. doi: 10.3171/2014.9.JNS14256 25361490

[38] Kajita Y, Nakatsubo D, Kataoka H, Nagai T, Nakura T, Wakabayashi T. Installation of a Neuromate Robot for Stereotactic Surgery: Efforts to Conform to Japanese Specifications and an Approach for Clinical Use-Technical Notes. Neurologia medico-chirurgica. 2015;55(12):907–914. doi: 10.2176/nmc.tn.2015-0043 26511113 PMC4686454

[39] Lefranc M, Peltier J. Evaluation of the ROSA™ Spine robot for minimally invasive surgical procedures. Expert Review of Medical Devices. 2016;13(10):899–906. doi: 10.1080/17434440.2016.1236680 27649314

[40] Cheng W, Adler JR. An overview of cyberknife radiosurgery. Chinese Journal of Clinical Oncology. 2006;3(4):229–243. doi: 10.1007/s11805-006-0049-5

[41] Iversen DH, Wein W, Lindseth F, Unsgård G, Reinertsen I. Automatic Intraoperative Correction of Brain Shift for Accurate Neuronavigation. World Neurosurgery. 2018;120:e1071–e1078. doi: 10.1016/j.wneu.2018.09.012 30213682

[42] Ries M, de Senneville BD, Roujol S, Berber Y, Quesson B, Moonen C. Real-time 3D target tracking in MRI guided focused ultrasound ablations in moving tissues. Magnetic Resonance in Medicine. 2010;64(6):1704–1712. doi: 10.1002/mrm.22548 20878763

[43] Clendenen SR, Candler SA, Osborne MD, Palmer SC, Duench S, Glynn L, et al. Needle placement for piriformis injection using 3-D imaging. Pain Physician. 2013;16(3):E301–310. doi: 10.36076/ppj.2013/16/E301 23703429

[44] Scholten HJ, Pourtaherian A, Mihajlovic N, Korsten HHM, Bouwman RA. Improving needle tip identification during ultrasound-guided procedures in anaesthetic practice. Anaesthesia. 2017;72(7):889–904. doi: 10.1111/anae.13921 28542716

[45] Kaya M, Denasi A, Scheggi S, Agbahca E, Yoon C, Gracias DH, et al. A Multi-Rate State Observer for Visual Tracking of Magnetic Micro-Agents Using 2D Slow Medical Imaging Modalities. In: 2018 IEEE/RSJ International Conference on Intelligent Robots and Systems (IROS); 2018. p. 1–8.

[46] Chevrie J, Shahriari N, Babel M, Krupa A, Misra S. Flexible Needle Steering in Moving Biological Tissue With Motion Compensation Using Ultrasound and Force Feedback. IEEE Robotics and Automation Letters. 2018;3(3):2338–2345. doi: 10.1109/LRA.2018.2809484

[47] Bhattacharji P, Moore W. Application of Real-Time 3D Navigation System in CT-Guided Percutaneous Interventional Procedures: A Feasibility Study. Radiology Research and Practice. 2017;2017:7. doi: 10.1155/2017/3151694 29181197 PMC5664284

[48] Ben-David E, Shochat M, Roth I, Nissenbaum I, Sosna J, Goldberg SN. Evaluation of a CT-Guided Robotic System for Precise Percutaneous Needle Insertion. Journal of Vascular and Interventional Radiology. 2018;29(10):1440–1446. doi: 10.1016/j.jvir.2018.01.002 29628297

[49] Gafford JB, Webster S, Dillon N, Blum E, Hendrick R, Maldonado F, et al. A Concentric Tube Robot System for Rigid Bronchoscopy: A Feasibility Study on Central Airway Obstruction Removal. Annals of Biomedical Engineering. 2020;48(1):181–191. doi: 10.1007/s10439-019-02325-x 31342337 PMC6930337

[50] Mehta AM, Sonabend AM, Bruce JN. Convection-Enhanced Delivery. Neurotherapeutics. 2017;14(2):358–371. doi: 10.1007/s13311-017-0520-4 28299724 PMC5398992

[51] Jamal A, Bernardini A, Dini D. Microscale characterisation of the time-dependent mechanical behaviour of brain white matter. Journal of the Mechanical Behavior of Biomedical Materials. 2022;125:104917. doi: 10.1016/j.jmbbm.2021.104917 34710852

[52] Vidotto M, Bernardini A, Trovatelli M, De Momi E, Dini D. On the microstructural origin of brain white matter hydraulic permeability. Proceedings of the National Academy of Sciences. 2021;118(36). doi: 10.1073/pnas.2105328118 34480003 PMC8433514

[53] Jamal A, Yuan T, Galvan S, Castellano A, Riva M, Secoli R, et al. Insights into Infusion-Based Targeted Drug Delivery in the Brain: Perspectives, Challenges and Opportunities. International Journal of Molecular Sciences. 2022;23(6). doi: 10.3390/ijms23063139 35328558 PMC8949870

[54] Secoli R, Rodriguez F, Baena. Experimental validation of curvature tracking with a programmable bevel-tip steerable needle. In: 2018 International Symposium on Medical Robotics (ISMR); 2018. p. 1–6.

[55] Watts T, Secoli R, Rodriguez y Baena F. Needle Steerability Measures: Definition and Application for Optimized Steering of the Programmable Bevel-Tip Needle. In: 2018 IEEE International Conference on Robotics and Biomimetics (ROBIO); 2018. p. 59–64.

[56] Gerovich O, Marayong P, Okamura AM. The effect of visual and haptic feedback on computer-assisted needle insertion. Computer Aided Surgery. 2004;9(6):243–249. 16112974 10.3109/10929080500190441

[57] Romano JM, Webster RJ, Okamura AM. Teleoperation of Steerable Needles. In: Proceedings 2007 IEEE International Conference on Robotics and Automation; 2007. p. 934–939.

[58] Pacchierotti C, Abayazid M, Misra S, Prattichizzo D. Teleoperation of Steerable Flexible Needles by Combining Kinesthetic and Vibratory Feedback. IEEE Transactions on Haptics. 2014;7(4):551–556. doi: 10.1109/TOH.2014.2360185 25265614

[59] Li WH, Liu B, Kosasih PB, Zhang XZ. A 2-DOF MR actuator joystick for virtual reality applications. Sensors and Actuators A: Physical. 2007;137(2):308–320. doi: 10.1016/j.sna.2007.03.015

[60] Matheson E, Secoli R, Galvan S, Rodriguez y Baena F. Human-Robot Visual Interface for 3D Steering of a Flexible, Bioinspired Needle for Neurosurgery. In: 2019 IEEE/RSJ International Conference on Intelligent Robots and Systems (IROS); 2019. p. 5426–5431.

[61] Ralovich K, John M, Camus E, Navab N, Heimann T. 6DoF catheter detection, application to intracardiac echocardiography. Medical image computing and computer-assisted intervention: MICCAI International Conference on Medical Image Computing and Computer-Assisted Intervention. 2014;17(Pt 2):635–642. 10.1007/978-3-319-10470-6_79 25485433

[62] Vrooijink GJ, Abayazid M, Misra S. Real-time three-dimensional flexible needle tracking using two-dimensional ultrasound. In: Proceedings—IEEE International Conference on Robotics and Automation. IEEE; 2013. p. 1688–1693.

[63] Chatelain P, Krupa A, Marchal M. Real-time needle detection and tracking using a visually servoed 3D ultrasound probe. In: 2013 IEEE International Conference on Robotics and Automation. IEEE; 2013. p. 1676–1681.

[64] Adebar TK, Fletcher AE, Okamura AM. 3-D Ultrasound-Guided Robotic Needle Steering in Biological Tissue. IEEE Transactions on Biomedical Engineering. 2014;61(12):2899–2910. doi: 10.1109/TBME.2014.2334309 25014948 PMC5545809

[65] Sadjadi H, Hashtrudi-Zaad K, Fichtinger G. Fusion of Electromagnetic Trackers to Improve Needle Deflection Estimation: Simulation Study. IEEE Transactions on Biomedical Engineering. 2013;60(10):2706–2715. doi: 10.1109/TBME.2013.2262658 23674421

[66] Shahriari N, Hekman E, Oudkerk M, Misra S. Design and evaluation of a computed tomography (CT)-compatible needle insertion device using an electromagnetic tracking system and CT images. International Journal of Computer Assisted Radiology and Surgery. 2015;10(11):1845–1852. doi: 10.1007/s11548-015-1176-3 25843947 PMC4617842

[67] Abayazid M, Moreira P, Shahriari N, Patil S, Alterovitz R, Misra S. Ultrasound-guided three-dimensional needle steering in biological tissue with curved surfaces. Medical Engineering and Physics. 2015;37(1):145–150. doi: 10.1016/j.medengphy.2014.10.005 25455165 PMC4409936

[68] Favaro A, Secoli R, Rodriguez y Baena F, DeMomi E. Model-Based Robust Pose Estimation for a Multi-Segment, Programmable Bevel-Tip Steerable Needle. IEEE Robotics and Automation Letters. 2020;5(4):6780–6787. doi: 10.1109/LRA.2020.3018406

[69] Khan F, Donder A, Galvan S, Baena FRy, Misra S. Pose Measurement of Flexible Medical Instruments Using Fiber Bragg Gratings in Multi-Core Fiber. IEEE Sensors Journal. 2020;20(18):10955–10962. doi: 10.1109/JSEN.2020.2993452

[70] Donder A, Rodriguez y Baena F. Kalman-Filter-Based, Dynamic 3-D Shape Reconstruction for Steerable Needles With Fiber Bragg Gratings in Multicore Fibers. IEEE Transactions on Robotics. 2021; p. 1–14.

[71] Pinzi M, Galvan S, Rodriguez y Baena F. The Adaptive Hermite Fractal Tree (AHFT): a novel surgical 3D path planning approach with curvature and heading constraints. International Journal of Computer Assisted Radiology and Surgery. 2019;. doi: 10.1007/s11548-019-01923-3 30790172 PMC6420904

[72] Zhan W, Tan Z, Bernardini A, Dini D, Rodriguez y Baena F. Effect of Enhanced Cerebrospinal Fluid Flow on Drug Penetration in Convection Enhanced Delivery. BioMedEng; 2019.

[73] Göbl R, Navab N, Hennersperger C. SUPRA: open-source software-defined ultrasound processing for real-time applications. International Journal of Computer Assisted Radiology and Surgery. 2018;13(6):759–767. doi: 10.1007/s11548-018-1750-6 29594853

[74] Pinzi M, Galvan S, Watts T, Secoli R, Rodriguez y Baena F. Path Replanning for Orientation-constrained Needle Steering. IEEE Transactions on Biomedical Engineering. 2021; p. 1–1. 33606622 10.1109/TBME.2021.3060470

[75] Secoli R, Rodriguez y Baena F. Adaptive path-following control for bio-inspired steerable needles. In: 2016 6th IEEE International Conference on Biomedical Robotics and Biomechatronics (BioRob); 2016. p. 87–93.

[76] Watts T, Secoli R, Rodriguez y Baena F. A Mechanics-Based Model for 3-D Steering of Programmable Bevel-Tip Needles. IEEE Transactions on Robotics. 2018;PP:1–16.

[77] Hanson AJ, Ma H. Parallel Transport Approach to Curve Framing; 1995.

[78] Selig JM. Geometric fundamentals of robotics. New York: Springer; 2005.

[79] Castellano A, Falini A. EDEN2020 Human Brain MRI Datasets for Healthy Volunteers; 2019. Available from: 10.5281/zenodo.3338449.

[80] Trovatelli M, Brizzola S, Zani DD, Castellano A, Mangili P, Riva M, et al. Development and in vivo assessment of a novel MRI-compatible headframe system for the ovine animal model. The International Journal of Medical Robotics and Computer Assisted Surgery. 2021;17(4):e2257. doi: 10.1002/rcs.2257 33817973

[81] Pieri V, Trovatelli M, Cadioli M, Zani DD, Brizzola S, Ravasio G, et al. In vivo Diffusion Tensor Magnetic Resonance Tractography of the Sheep Brain: An Atlas of the Ovine White Matter Fiber Bundles. Frontiers in Veterinary Science. 2019;6:345. doi: 10.3389/fvets.2019.00345 31681805 PMC6805705

[82] Castellano A, Pieri V, Falini A. EDEN2020 Ovine Diffusion Tensor Magnetic Resonance Tractography Atlas; 2019. Available from: 10.5281/zenodo.5715981.

[83] Marcus HJ, Vakharia VN, Ourselin S, Duncan J, Tisdall M, Aquilina K. Robot-assisted stereotactic brain biopsy: systematic review and bibliometric analysis. Child’s Nervous System. 2018;34(7):1299–1309. doi: 10.1007/s00381-018-3821-y 29744625 PMC5996011

